# Global mRNA and miRNA Analysis Reveal Key Processes in the Initial Response to Infection with WSSV in the Pacific Whiteleg Shrimp

**DOI:** 10.3390/v13061140

**Published:** 2021-06-13

**Authors:** Rebecca S. Millard, Lisa K. Bickley, Kelly S. Bateman, Audrey Farbos, Diana Minardi, Karen Moore, Stuart H. Ross, Grant D. Stentiford, Charles R. Tyler, Ronny van Aerle, Eduarda M. Santos

**Affiliations:** 1 Biosciences, College of Life and Environmental Sciences, University of Exeter, Exeter EX4 4QD, UK; L.K.Bickley@exeter.ac.uk (L.K.B.); C.R.Tyler@exeter.ac.uk (C.R.T.); 2 Centre for Sustainable Aquaculture Futures, University of Exeter, Exeter EX4 4QD, UK; kelly.bateman@cefas.co.uk (K.S.B.); stuart.ross@cefas.co.uk (S.H.R.); grant.stentiford@cefas.co.uk (G.D.S.); ronny.vanaerle@cefas.co.uk (R.v.A.); 3 Cefas Weymouth Laboratory, International Centre of Excellence for Aquatic Animal Health, Weymouth DT4 8UB, UK; diana.minardi@cefas.co.uk; 4 Exeter Sequencing Service, Geoffrey Pope Building, University of Exeter, Exeter EX4 4QD, UK; A.Farbos@exeter.ac.uk (A.F.); K.A.Moore@exeter.ac.uk (K.M.)

**Keywords:** aquaculture, invertebrate, transcriptome, RNA-seq

## Abstract

White Spot Disease (WSD) presents a major barrier to penaeid shrimp production. Mechanisms underlying White Spot Syndrome Virus (WSSV) susceptibility in penaeids are poorly understood due to limited information related to early infection. We investigated mRNA and miRNA transcription in *Penaeus vannamei* over 36 h following infection. Over this time course, 6192 transcripts and 27 miRNAs were differentially expressed—with limited differential expression from 3–12 h post injection (hpi) and a more significant transcriptional response associated with the onset of disease symptoms (24 hpi). During early infection, regulated processes included cytoskeletal remodelling and alterations in phagocytic activity that may assist WSSV entry and translocation, novel miRNA-induced metabolic shifts, and the downregulation of ATP-dependent proton transporter subunits that may impair cellular recycling. During later infection, uncoupling of the electron transport chain may drive cellular dysfunction and lead to high mortalities in infected penaeids. We propose that post-transcriptional silencing of the immune priming gene Dscam (downregulated following infections) by a novel shrimp miRNA (Pva-pmiR-78; upregulated) as a potential mechanism preventing future recognition of WSSV that may be suppressed in surviving shrimp. Our findings improve our understanding of WSD pathogenesis in *P. vannamei* and provide potential avenues for future development of prophylactics and treatments.

## 1. Introduction

Aquaculture is an important industry for global food security. Farmed crustaceans currently account for approximately 11% of total aquaculture production by volume and 28% by value [1]. Penaeid shrimp register highly in terms of quantity and value; the Pacific whiteleg shrimp (*Penaeus vannamei*) being the most widely farmed species in an industry valued at approximately US$30 billion [1]. Most shrimp are farmed at high density and there are continued efforts to intensify this further. This intensification, and the regular and extensive movement of shrimp larvae and broodstock between aquaculture sites, have resulted in the rapid emergence and spread of pathogens. Losses due to disease may exceed 40% of global capacity annually [2]. Consequently, disease is considered one of the greatest factors limiting productivity and future expansion of shrimp aquaculture [3] with approximately 60% of these losses being attributable to viral diseases [4].

White Spot Syndrome Virus (WSSV) has inflicted an estimated US$21 billion loss in the farmed shrimp industry since its emergence in 1992 [2]. The virus spreads swiftly through aquatic ecosystems and international trade has facilitated its spread to all major shrimp farming areas of the world. Despite significant research efforts on WSSV, effective treatments are not yet widely available to limit its impact. Following infection with WSSV, progression to disease occurs rapidly (3–10 days), leading to high mortality (90–100%) in production systems [5].

Understanding the molecular interactions between the host and the pathogen following infection, particularly during the early stages, could aid in the development of White Spot Disease (WSD) treatments and prophylactics. Applying sequencing technologies to the study of WSSV pathogenesis enables insights into the changes of both the viral and host genomes and facilitates understanding of host–pathogen interactions. Multiple WSSV genomes have been sequenced [6,7,8,9,10,11,12,13], which have enhanced our understanding of WSSV gene expression, genome evolution, and disease epidemiology. Next-generation sequencing has been applied to study the responses of penaeid shrimp to WSSV infection with decreasing costs, increasing the technology’s accessibility [14]. Currently, transcriptomic data associated with WSSV infection are available for *P. vannamei* [15,16,17,18,19], Kuruma prawn *Penaeus japonicus* [20], and Chinese shrimp *Penaeus chinensis* [21,22]. These studies have contributed to our understanding of the transcriptional responses during acute and latent infections, upon imminent death, and in response to combined infection and temperature stressors. An initial study into the temporal dynamics of global gene transcription during WSSV infection reported dynamic, time-dependent alterations of cytoskeletal, metabolic, immune, and apoptotic processes at 1.5, 18, and 54 h post injection (hpi) [17]. Despite this, our understanding, both when considering the gills—one of the initial sites of infection of WSSV in shrimp [23]—and the early hours/phase of WSSV infection, remains limited.

Invertebrate miRNAs form a key component of the innate immune system and antiviral response [24], and are also widely recognised as important factors mediating the progression of disease [25,26,27,28], including the replication of viruses [29,30,31,32,33]. The miRNA responses of multiple crustacean species including *P. japonicus* [34], *P. chinensis* [35], *Penaeus monodon* [36], *P. vannamei* [37,38,39], *Cherax quadricarinatus* [40], and *Procambarus clarkii* [41] to WSSV have been studied. Despite this, miRNA expression profiles of the gill tissue, which is a primary site of WSSV infection, has been largely overlooked until recently [37,39] and critically, previously published studies lack detailed temporal information regarding the miRNA expression changes in response to very early WSSV infection.

Here we sought to further the molecular understanding of the host–pathogen interactions between WSSV and *P. vannamei* through studying the temporal dynamics of global transcriptional changes, both mRNA and miRNA, following WSSV infection. In our approach (encompassing temporal changes from the early stages of infection; adopting the recommended specific-pathogen-free (SPF) homogenate control injections [16]; and without the need for pooling individual samples for sequencing [42]), we employ RNA sequencing technology to generate a detailed global transcriptome from the gills of WSSV-infected *P. vannamei*, with mRNA and miRNA transcriptional datasets derived from the same biological samples. Our results reveal transcriptomic responses occurring in the earliest stages of interaction between WSSV and its host.

## 2. Materials and Methods

### 2.1. Virus Inoculum Preparation

WSSV isolate (UAZ 00-173B), from the University of Arizona, Tucson, Arizona, USA, OIE reference laboratory for WSSV, that retained virulence through passages in specific-pathogen-free (SPF) *P. vannamei* at Cefas Weymouth laboratory was used. Inoculums were generated as previously described [43] by passaging WSSV in *P. vannamei*, and homogenising uninfected and WSSV-infected shrimp in 2% sterile saline to generate SPF- and WSSV-homogenates (infection was confirmed using histopathology and nested PCR). Homogenates were centrifuged at 5000× *g* for 20 min at 4 °C to pellet cellular debris and the supernatants were diluted 1:20 in sterile saline and filtered using a 0.2 μm syringe filter (Sartorius Stedim Biotech GmbH (Göttingen, Germany)). The WSSV inoculum contained approximately 2.72 × 10^7^ virions/mg (quantified by qPCR).

### 2.2. Disease Trial

**Figure 1:**
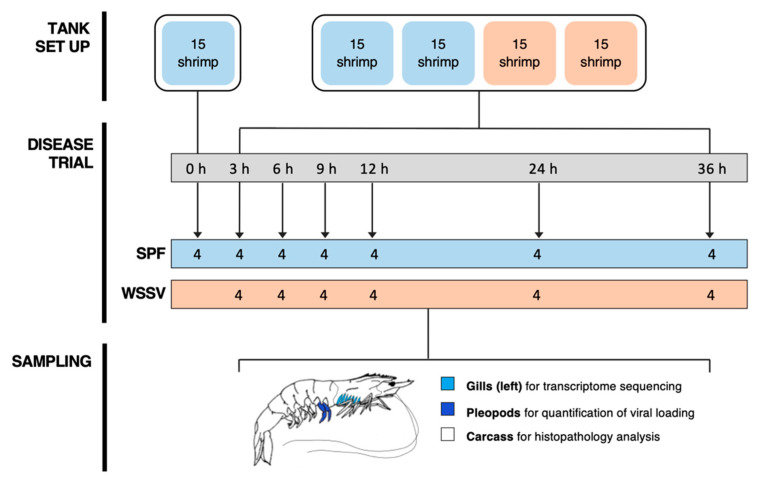
Schematic of the WSSV injection trial experimental set-up and sampling. Pacific whiteleg shrimp were randomly allocated into two groups. At zero hours, four control shrimp were sampled from a separate tank to provide a time-zero histological control reference. The remaining shrimp were injected with either specific-pathogen-free (SPF) or WSSV-infected shrimp inoculum. Four shrimp (two per treatment tank) were sampled from each treatment group at subsequent timepoints: 3, 6, 9, 12, 24, and 36 h. At each sampling point, gills from the left side of the shrimp were dissected and snap-frozen for transcriptome sequencing. The two most anterior pleopods were sampled to quantify viral loading, and the remaining carcass was fixed for histological analysis.

Sixty shrimp (5.0 ± 1.5 g standard deviation) were divided into two treatment groups and injected with either SPF or WSSV-infected shrimp inoculum at a dosage of 10 μL g^−1^ (wet body weight). This corresponded to an average of 6.80 × 10^7^ virions per shrimp. Four unexposed shrimp from an additional control tank were sampled at 0 h to provide a histological control independent of the disease trial. Shrimp were regularly checked throughout the disease trial and moribund shrimp removed to limit virus transmission via cannibalism. Two shrimp were sampled from each tank at 3, 6, 9, 12, 24, and 36 h post injection (hpi) totalling 4 shrimp per treatment and timepoint (Figure 1). Shrimp were euthanised on ice for 10 min prior to dissection in which the gills from the left-hand side of the shrimp were removed, snap-frozen in liquid nitrogen, and stored at −80 °C for transcriptome profiling. Two anterior pleopods were removed and stored in absolute ethanol for quantification of viral load and the remaining carcasses were fixed immediately for histopathology analysis by injecting Davidson’s seawater fixative at regular intervals along their lengths and submerging them in fixative for 24 h.

### 2.3. Infection Confirmation and Viral Load Quantification

To confirm the presence/absence of WSSV infection in each treatment group, histopathological examination was performed. Following fixation, shrimp carcasses were transferred to 70% IMS and sectioned in order to fit within standard histology cassettes and reveal the main organs of interest (gill, gut, and cuticular epithelium). Samples were further processed according to previous standard methods [43]. Viral load was quantified by qPCR on DNA extracted from pleopods (20–45 mg) using the EZ1 DNA Tissue kit and BioRobot^®^ EZ1 (Qiagen (Hilden, Germany)). Briefly, pleopods were homogenised in 1:10 *w/v* tissue to G2 buffer (Qiagen (Hilden, Germany)) with Lysing Matrix FastPrep^®^ A tubes and a FastPrep^®^ cell disrupter (1 min at 5 m/s), followed by overnight digestion with proteinase K (10 µL at 10 µg/mL) at 56 °C. qPCR conditions and positive control plasmid preparation followed previously published methods [44], using 20 µL reactions and 2.5 µL of DNA. Reactions were performed in triplicates and included negative controls with molecular grade water instead of DNA. Quantification was performed by generating standard curves with dilution series of 4 × 10^7^ copies/μL of plasmids. qPCR reactions were performed on an ABI Biosystems TaqMan machine and copy numbers calculated with StepOne™ Software v2.3 (Applied Biosystems (Waltham, MA, USA)).

### 2.4. RNA Extraction, Library Preparation, and Sequencing

Total RNA was extracted from gills (n = 48) using the miRNeasy Mini Kit (Qiagen (Hilden, Germany)) with on-column DNase I (Qiagen (Hilden, Germany)) treatment to remove DNA contaminants. RNA quality was assessed via Tapestation using the RNA Analysis ScreenTape System (Agilent (Santa Clara, CA, USA)). mRNA libraries were prepared using the TruSeq stranded mRNA sample preparation kit with TruSeq A and B adapter sets (Illumina (San Diego, CA, USA)) to enable multiplexing of the 48 libraries into two pools. An equal number of control and WSSV-injected samples from each timepoint were randomly allocated to each library pool. These were sequenced on an Illumina HiSeq 2500 to 100 bp in paired-end mode. miRNA-seq libraries were constructed using the NEXTFlex^TM^ small RNA sequencing kit v3 (Bioo Scientific Corporation (Austin, TX, USA)) with 18 PCR amplification cycles and gel-free size selection protocol. The final pooled library was size selected using the Sage Pippin Prep System (Sage Science (Beverly, MA, USA)) and sequenced to 50 bp in single read mode on the Illumina HiSeq 2500.

### 2.5. Transcriptome Analysis

Detailed steps for transcriptomic analysis are given in Figure S1. Briefly, raw reads were subject to quality assessments and trimmed with trimmomatic v0.36 [45] to remove adapters, short reads, and low-quality bases. Trinity v2.4.0 [46] was used to de novo assemble the transcriptome, and transcript redundancy was reduced with CD-HIT-EST v4.6 [47]. Transcriptome assembly metrics including mean contig length, Ex90N50, BUSCO (v2.0) scores [48], and read representation were used to assess the quality of the assembly. Transcripts were annotated using the BlastX algorithm of Diamond v0.8.27 [49] and the RefSeq Release 85 protein database using an e-value cut-off of 1 × 10^−5^ with sensitive mode enabled. Hits were filtered taxonomically using MEGAN Community Edition v6.6.0 [50] and transcripts from metazoan and viral clades, which were assumed to have arisen from the host and the virus, respectively, were extracted. Trimmed reads were then aligned to the filtered transcriptome using Bowtie v1.1.2 [51] and transcript abundance was estimated using RSEM v1.3.0 [52]. Differentially expressed transcripts were identified by comparing WSSV-injected shrimp with their time matched controls using edgeR v3.16.3 [53] with a false discovery rate (FDR) cut-off of 0.05 [54]. Trends in the dataset were identified by principal component analysis using the Trinity PtR script with the complete transcript counts table. Gene ontology (GO) terms were assigned to transcripts within Blast2Go PRO v5.0 [55] and enriched GO terms identified using Gene Set Enrichment Analysis (GSEA) software [56] with 1000 permutations, minimum gene set size of two, and a significance cut-off of 0.05. Kyoto Encyclopaedia of Genes and Genomes (KEGG) pathway annotations were retrieved by inputting *Drosophila melanogaster* transcript homologs identified by a Diamond BlastX search of differentially expressed shrimp transcripts against the Uniprot *D. melanogaster* complete proteome (e-value 1 × 10^−20^) into the KEGG Automatic Annotation Server (KAAS) v2.1 (release 86.1) [57] with bi-directional best hits. KEGG pathway representation was then assessed using the Generally Applicable Gene-set/Pathway Analysis (GAGE) package v3.9 [58] in R v3.6 [59] with *D. melanogaster* dataset as background and a significance cut-off of 0.05. Expression data were overlaid on KEGG pathways using Pathview v3.9 [60]. Finally, the presence of the replicating virus was detected in each sample by aligning trimmed reads against the WSSV-CN (GenBank Accession: AF332093.3 [61]) genome sequence using Bowtie2 [62]. Where more than 1% of the total sequencing reads were aligned to WSSV-CN, the virus was deemed to be present and replicating.

### 2.6. miRNA Analysis

A detailed description of tools and parameters used in the analysis of small RNA sequences is provided in Figure S2. Raw small RNA sequences were quality trimmed to remove adapter sequences (including randomised 4 nt adapter signatures incorporated during library preparation), and sequences outside the expected length range of miRNAs (18–26 nt) using Cutadapt v2.1 [63]. Trimmed reads aligned to the *P. vannamei* ribosomal RNA sequence (GenBank Accession: AF124597.1) with Bowtie v1.0.0 [51] were removed using Samtools v1.4 [64] and Seqtk v1.2-r94 [65]. Reads with more than 50% of their lengths comprised of low complexity sequences (e.g., homopolymers) were also removed using the Fqtrim v0.9.7 DUST filter [66]. The miRDeep2 v2.0.0.8 [67] pipeline was employed to collapse reads, align reads to the *P. vannamei* genome (GenBank Accession: QCYY00000000.1 [68]), predict, and quantify miRNAs. miRNA predictions were guided by mature miRNA sequences from *Tribolium castaneum* (n = 211) from the miRGeneDB database [69] and miRNAs identified in closely related *Penaeus japonicus* (n = 63) [70]. miRNAs with Rfam hits were removed and the redundancy was reduced using CD-HIT-EST v4.8.1 [47] prior to quantification. miRNAs were annotated using BlastN [71] optimised for sequences < 30 nt with an e-value of 1 × 10^−5^ against the previously described *P. vannamei* miRNAs [72], *P. japonicus* miRNAs [70], as well as *T. castaneum*, *D. melanogaster*, and *Daphnia pulex* miRNAs from the miRGeneDB database [69]. Due to the highly conserved nature of miRNAs, predicted miRNAs with hits were classified as known homologs whilst those with no annotation were considered novel. Novel *P. vannamei* miRNAs were labelled as putative miRNAs with ‘pmiR’ and assigned a unique number. Differentially expressed miRNAs were identified between WSSV-injected shrimp and time-matched controls using DESeq2 v3.9 [73] with a Benjamin–Hochberg-adjusted significance cut-off of 0.05 [54]. miRNA secondary structures were predicted using RNAfold v2.4.11 [74] and their folding stability was predicted with randfold [75]. Details of the WSSV miRNA analyses are specified within Appendix A.

### 2.7. Integrated Analysis of RNA-Seq and miRNA-Seq

To determine the functionality of differentially expressed miRNAs, the ability of these to bind to transcripts within the assembled transcriptome was predicted using miRanda v3.3a [76] and RNA22 v2 [77] specifying strict alignment in the seed region and a minimum free energy score less than −20 kcal/mol. As per recommended best practice [78], interactions that were predicted by both tools were considered potential targets. Enriched GO terms were identified within the lists of predicted target transcripts for each differentially expressed miRNA within Blast2Go PRO v5.0 [55] using a Fisher’s Exact test with an adjusted significance cut-off of 0.05. The relative expression of differentially expressed miRNAs and target transcripts of interest that were associated with significantly enriched GO terms was plotted and compared. Plots were generated in R using ggplot2 [79]; all error bars represent ± one standard error of the mean.

## 3. Results

### 3.1. Disease Presentation

**Figure 2:**
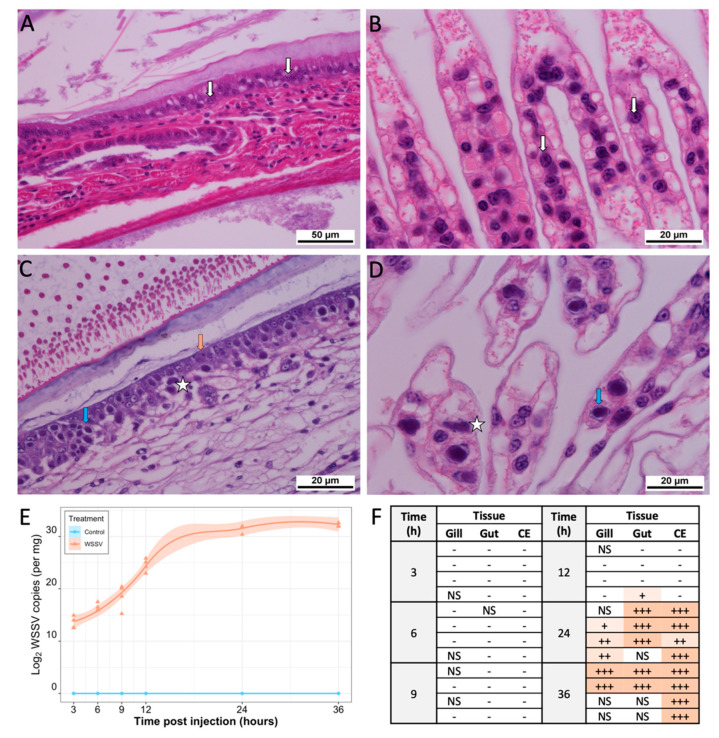
Phenotypic evidence for infection status in control and WSSV-injected treatments. Shrimp from control and WSSV-injected groups were subjected to histological screening, using haematoxylin and eosin staining with light microscopy, for signs of WSSV infection in the gill, gut, and cuticular epithelium tissue. (**A**) Control gut and (**B**) control gill tissues contain small, regularly shaped nuclei with basophilic stain (white arrow). (**C**) Nuclei infected with WSSV in the gut and (**D**) gills were enlarged. During early infection their chromatin was marginalised and large inclusion bodies stained homogeneous eosinophilic (orange arrow). As the infection progressed, these inclusions became increasingly basophilic (blue arrow) before disintegrating so that the contents fused with the cytoplasm (left of white star). (**E**) Scatter plot with smoothed conditional local regression line depicting log2 WSSV copy number over time, measured by qPCR. Shaded areas represent 95% confidence interval. (**F**) Table depicting the infection status of gill, gut, and cuticular epithelium (CE) tissue of WSSV-injected shrimp at each timepoint following WSSV injection. No infection is denoted by “-”, mild infection (few enlarged nuclei present) by “+”, moderate infection (at least 50% of cells within the target tissue present enlarged nuclei) by “++”, and severe infection (over 80% of the cells within the target tissue present enlarged nuclei) by “+++”. Tissues that were not able to be sectioned are marked “NS”.

Control shrimp remained apparently healthy throughout the experiment, displaying no visible signs of disease. However, two shrimp (one at 12 hpi and another at 24 hpi) died during the study, likely attributable to handling stress following the sham injection. In the WSSV treatment group, shrimp displayed histopathological symptoms of infection from 24 hpi, with mortalities occurring at 24 hpi (n = 3) and at 36 hpi (n = 3), which coincided with an exponential increase in viral copy number and pathognomonic signs of disease observed with histopathology (Figure 2).

### 3.2. Sequencing and Transcriptome Assembly

Sequencing produced 698,628,573 paired-end reads (2 × 100 bp), averaging 14,554,762 read pairs per individual shrimp sample. A transcriptome was de novo assembled using 660,175,443 quality-trimmed reads to produce 116,801 reduced-redundancy transcripts (87,774 loci). The transcriptome had an N50 of 1255 bp, Ex90N50 of 2220 bp, mean transcript length of 760 bp, and on average 98.0% of the reads from each sample were represented within the transcriptome. In addition, the present transcriptome was near-complete, containing 97.7% of Arthropoda BUSCO’s as either single (80.3%) or duplicated copies (17.4%) with few present as fragments (0.8%) or missing (1.5%). The RefSeq complete protein database (release 85) was used to assign annotations to 25,813 (22.1%) transcripts, which were subsequently filtered to retain only Metazoan (23,879) and Viral (194) transcripts assumed to have derived from the host and from WSSV, respectively. Within the Metazoan taxon, the Pancrustacea (14,619) were the most strongly represented group.

### 3.3. Transcript Expression Analysis

**Figure 3:**
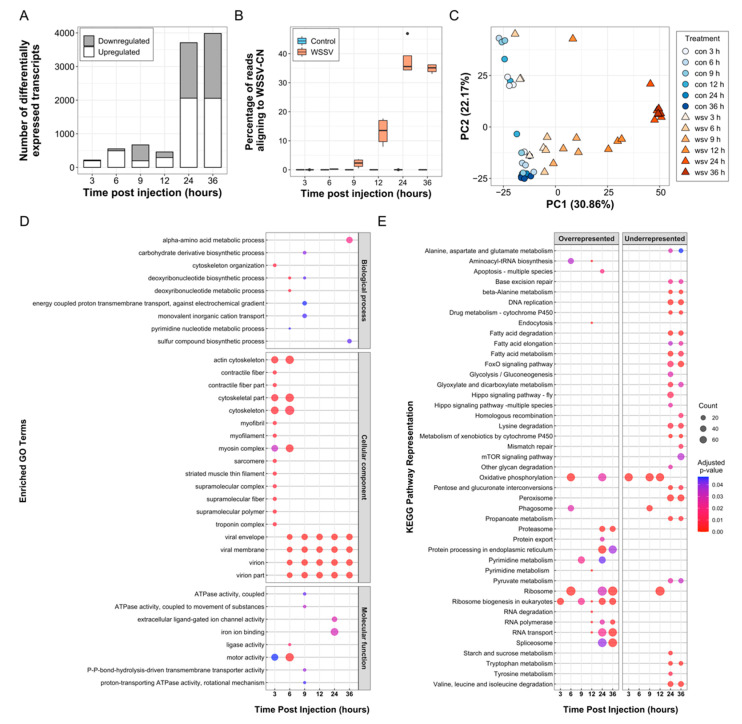
Transcriptome analysis. (**A**) A stacked bar plot depicting the number of significantly upregulated and downregulated transcripts over time following WSSV injection. (**B**) Boxplot illustrating the percentage of trimmed sequencing reads from control and WSSV-injected shrimp aligning to the WSSV-CN genome (GenBank Accession: AF332093.3 [61]) over time. (**C**) Principal component analysis demonstrating similarity between the differentially expressed transcripts in each treatment and timepoint according to principal component 1 (PC1) and principal component 2 (PC2). (**D**) Temporal enrichment of gene ontology (GO) terms within the lists of differentially expressed transcripts at each timepoint following WSSV injection. The size of each point indicates the number of transcripts assigned to each GO term, and the colour indicates the adjusted *p*-value associated with the GO term’s enrichment. (**E**) Over- and underrepresented KEGG pathways within the expressed transcripts at each timepoint following WSSV injection. Point size indicates the number of transcripts present within each KEGG pathway, and the colour indicates the adjusted *p*-value associated with the KEGG pathway’s enrichment.

In total, 6192 transcripts were differentially expressed in at least one timepoint between WSSV and the time-matched controls. The number of differentially expressed transcripts followed a biphasic pattern with low numbers during very early infection (≤670 per timepoint from 3–12 hpi) and substantially higher numbers during later infection (≥3709 per timepoint at 24 and 36 hpi) (Figure 3A). Large transcriptional changes during later timepoints coincided with the appearance of clinical signs of disease and rapid onset of mortality (Figure 2). In addition, the proportion of WSSV-derived sequences in WSSV-injected shrimp increased from 0.03% (3 hpi) to 13.2% (12 hpi), reaching a peak of 38.1% of transcripts at 24 hpi (Figure 3B). A principal component analysis (PCA) of differentially expressed transcripts demonstrated minimal variation in control treatments that clustered together in the first principal component (PC1) (Figure 3C). In contrast, WSSV-injected shrimp separated along PC1 according to time post injection, except for late timepoints (24 and 36 hpi), the differential expression profiles of which were not sufficiently different to cluster separately. A large amount of variation attributed to PC2 was also observed, likely due to the effect of handling animals during injection—highlighting the importance of time-matched controls. A full list of differentially expressed transcripts is presented in Datasets S1 to S6.

### 3.4. miRNA Sequencing, Prediction and Analysis

**Figure 4:**
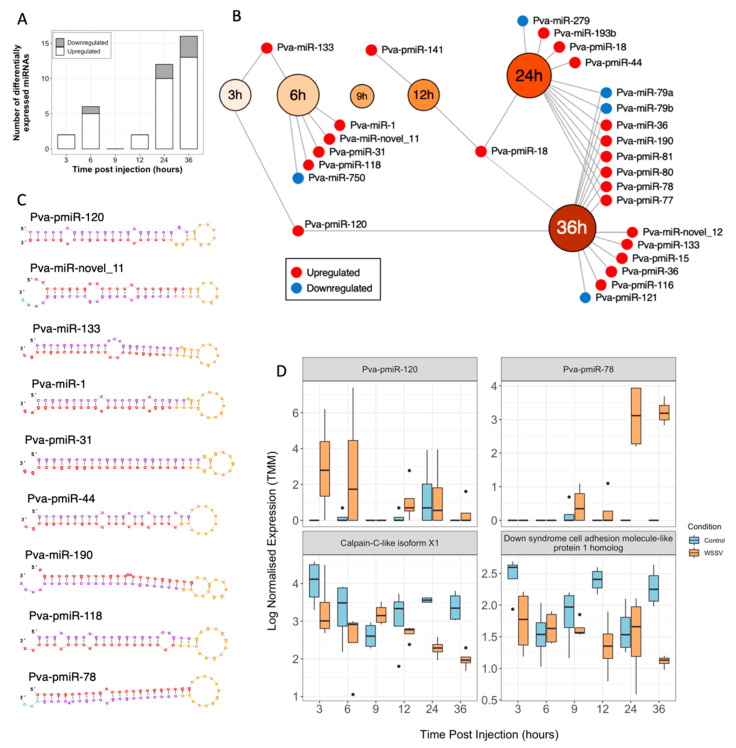
miRNA-seq results. (**A**) Stacked bar plot displaying the number of significantly differentially expressed miRNAs at each timepoint following WSSV injection. (**B**) Venn diagram depicting the overlap in differentially expressed miRNAs between the timepoints sampled following WSSV injection. (**C**) RNAfold structure of the miRNAs discussed herein. Red nucleotides depict the mature miRNA sequence, yellow nucleotides correspond to the characteristic hairpin loop of miRNA precursors and purple nucleotides depict the star miRNA sequence. (**D**) Boxplots displaying the temporal transcription patterns of novel shrimp miRNA Pva-pmiR-120, which is significantly upregulated at 3 hpi, and its predicted target calpain-C-like isoform X1, which is subsequently downregulated. In addition, a boxplot of novel shrimp miRNA Pva-pmiR-78, which is significantly upregulated at 24 and 36 hpi, and its predicted target down syndrome cell adhesion molecular-like protein 1 homolog, which is concomitantly downregulated.

A total of 120,409,899 small RNA sequencing reads were obtained. Quality trimming and filtering resulted in retention of 33.9% of the total reads (40,773,190), averaging 849,441 (±65,170) reads per sample, for downstream analyses. Condensation of these reads revealed 1,976,755 unique sequences across the 48 samples, with an average of 44,182 (±3309) unique sequences per sample. In addition, 655,964 alignments to the shrimp genome were reported for prediction of 296 reduced-redundancy *P. vannamei* miRNAs using the miRDeep2 pipeline (Dataset S7). Sixty-three (21.3%) were assigned annotations and 233 (78.7%) were considered novel. Differential expression analysis revealed 27 WSSV-responsive shrimp miRNAs that were significantly differentially expressed in at least one timepoint following injection (Table S1). Similar to the transcriptome, the number of significantly differentially expressed miRNAs followed a biphasic expression pattern, with limited differential expression in the very early stages (≤6) compared to later stages of infection (≥13) (Figure 4A,B). In addition, most of the differentially expressed miRNAs were upregulated (22, 81.5%) in response to WSSV injection.

### 3.5. Functional Analysis of Transcriptome and miRNA Data

Functional analysis of differentially expressed transcripts identified 35 significantly enriched GO terms (*p* < 0.05) (Figure 3D) and 43 significantly over- or underrepresented KEGG pathways (*p* < 0.05) (Figure 3E) throughout the infection process—these are listed in Tables S2 and S3. At each timepoint, distinct processes were altered in response to WSSV infection. Among the differentially expressed miRNAs, 1729 unique target transcripts were predicted in the transcriptome (Dataset S8), and across all miRNA target lists 480 GO terms were enriched, indicating a broad array of processes that may be regulated by the WSSV-responsive miRNAs detected.

#### 3.5.1. Cytoskeleton Remodelling Assists Early WSSV Entry and Movement

From 3–6 hpi, the most significantly enriched terms were associated with components of the cytoskeleton and the process of cytoskeleton organization (Figure 3D). The contributing transcripts included both cytoskeletal building blocks (e.g., Actin-57B (3 hpi: logFC = 9.43, FDR = 3.01 × 10^−3^, 6 hpi: logFC = 11.20, FDR = 2.65 × 10^−3^) and Actin-1-like (3 hpi: logFC 10.24, FDR = 7.20 × 10^−6^, 6 hpi: logFC = 10.54, FDR = 5.50 × 10^−5^)) and components of the molecular motors that enable intracellular movement (e.g., 9 myosin heavy chain isoforms including myosin heavy chain, and muscle-like isoforms X2 (3 hpi: logFC = 11.72, FDR = 4.80 × 10^−5^, 6 hpi: logFC = 9.62, FDR = 1.97 × 10^−4^) and X8 (3 hpi: logFC = 10.36, FDR = 2.74 × 10^−6^, 6 hpi: logFC = 13.00, FDR = 2.26 × 10^−5^)), which were significantly upregulated in response to WSSV injection.

Concomitantly, novel miRNAs Pva-pmiR-120 (3hpi: logFC = 22.65, FDR = 3.45 × 10^−7^) and Pva-miR-novel_11 (6hpi: logFC = 0.98, FDR = 0.02) (Figure 4C), with lengths of 19 and 22 nt, randfold (1.6 and 1.6) and minimum free energy (MFE) (2.2 and 2.1) values supporting their validity as true miRNAs, were significantly upregulated (Figure 4D). For each of these, many mRNA targets were predicted (141 and 201, respectively). The biological function of Pva-pmiR-120 may be inferred by the enrichment of terms in the target transcript list for the regulation of microtubule polymerisation or depolymerisation (FDR = 7.74 × 10^−4^) and calcium-dependent cysteine-type endopeptidase activity (FDR = 2.80 × 10^−3^). This is further supported by the fact that this miRNA is predicted to target the transcripts tubulin-specific chaperone cofactor E-like protein (required for correct organisation of microtubule cytoskeleton) and calpain C-like isoforms (involved in breakdown of cytoskeletal proteins and cytoskeletal remodelling), respectively (Figure 4D). In addition, Pva-miR-novel_11 was predicted to target myosin-11-like isoform X2 among other cytoskeletal transcripts, suggesting a role for these miRNAs in cytoskeletal remodelling during early WSSV infection. These hypotheses are based on bioinformatics predictions and require further validation work to demonstrate the biological significance of these predicted interactions.

#### 3.5.2. Pathogen-Associated Phagocytic Activity Is Altered during Early Infection

Two previously described miRNAs, Pva-miR-133 (3 hpi: logFC = 6.41, FDR = 2.17 × 10^−3^, 6 hpi: logFC = 8.00, FDR = 1.76 × 10^−5^) and Pva-miR-1 (6 hpi: logFC = 2.70, FDR = 1.43 × 10^−3^) (Figure 4C) were upregulated during early infection. Pva-miR-133 was predicted to interact with 167 transcripts that were enriched for GO terms SWI/SNF complex (FDR = 5.26 × 10^−3^) and ATP-dependent chromatin remodelling (FDR = 3.42 × 10^−2^). The SWI/SNF-related matrix-associated actin-dependent regulator of chromatin subfamily E member 1-like isoform X2 which contributed to this enrichment, was downregulated at 9 hpi (logFC = −4.27, *p* = 4.63 × 10^−2^), and plays a fundamental role in the regulation of phagocytosis, chromatin remodelling and transcriptional regulation [80,81]. Pva-miR-1 target, the regulator of G-protein signalling 9 isoform X2, which plays a functional role in the regulation of Ras and Rho signal transduction, may also regulate phagocytosis during early WSSV infection. This is further supported by the significant overrepresentation of the phagosome pathway (KEGG ID: dme04145) at 6 hpi (FDR = 0.03) and subsequent underrepresentation at 9 hpi (FDR = 2.15 × 10^−4^).

#### 3.5.3. WSSV Replication First Detected at 6 hpi

The enrichment of the deoxyribonucleotide metabolic process and deoxyribonucleotide biosynthetic process terms at 6 hpi indicated an increase in DNA replication (Figure 3D). This coincided with the upregulation of virus-derived transcripts such as thymidylate synthetase (WSV067) (logCPM = 4.74), TK-TMK chimeric thymidine and thymidylate kinase (WSV395) (logCPM = 3.98), and viral DNA polymerase (WSV514) (logCPM = 4.40), which provide considerable independence from the host’s nucleotide metabolism machinery. These transcripts also contributed to the significant enrichment of virus-associated GO terms virion part, virion, viral envelope, and viral membrane (FDR < 0.01) during this timepoint. Host transcription-associated mediator of RNA polymerase II transcription subunit 15-like was significantly upregulated (logFC = 10.10, FDR = 1.20 × 10^−5^) and the ribosome KEGG pathway overrepresented (Figure 3E) at 6 hpi, suggesting an accompanying increase in protein biosynthesis at this timepoint.

#### 3.5.4. Metabolic Shifts Associated with a White Spot-Induced Warburg Effect Are Likely Driven by Novel WSSV-Responsive miRNAs

Novel shrimp miRNAs Pva-pmiR-31 and Pva-miR-novel-11, which were significantly upregulated at 6 hpi (logFC = 1.74, FDR = 1.71 × 10^−4^ and logFC = 0.98, FDR = 0.02, respectively), were predicted to target 6 and 201 mRNAs, respectively. These lists of mRNA targets were enriched for metabolism-associated GO terms such as 2,6-bisphosphate metabolic process (FDR = 6.55 × 10^−3^) and phosphopyruvate hydratase activity (FDR = 5.19 × 10^−10^). The mRNAs contributing to these terms included enzymes such as 6-phosphofructo-2-kinase/fructose-2,6-bisphosphatase and enolase isoforms X1 and X2, which play a role in determining the rates of glycolysis and may play a role in establishing the Warburg-like effect associated with metabolic changes in response to WSSV infection [82,83].

#### 3.5.5. ATP-Dependent Proton Transporter Downregulation May Disrupt Lysosome Formation at 9 hpi

As infection progressed, terms associated with PP-bond-hydrolysis-driven transmembrane transporter activity and ATPase activity, coupled with transmembrane movement of substances, were enriched (Figure 3D). This enrichment was associated with a significant downregulation of 12 V-type ATPase subunits, including the 16 kDa proteolipid subunit (logFC = −5.36, FDR = 1.16 × 10^−3^) and subunits C-, D-, and E-like (logFC = −3.65, FDR = 2.22 × 10^−4^, logFC = −3.53, FDR = 6.03 × 10^−4^, logFC = −3.24, FDR = −1.85 × 10^−3^, respectively). These membrane-embedded ATPases are associated with vesicle acidification, therefore their downregulation has the potential to subvert lysosome formation to benefit either WSSV or the host. This also has a significant impact on the phagosome pathway, which was significantly underrepresented at 9 hpi (Figure 3E).

#### 3.5.6. Oxidoreductase Activity Was Strongly Perturbed at 24 hpi in WSSV-Infected Shrimp

At 24 hpi, significant alterations to the transcription of electron transport chain components may associate with oxidative stress, explaining the mechanism by which WSSV infection causes cell death and mortality. This was supported by the enrichment of oxidoreductase activity terms (acting on paired donors, with incorporation or reduction of molecular oxygen, and acting on CH-NH groups of donors, NAD or NADP as acceptor (Figure 3D)), and also by the overrepresentation of the oxidative phosphorylation KEGG pathway at 24 hpi (Figure 3E). Overlaying the expression data on this KEGG pathway (Figure S3) demonstrated temporal shifts throughout the experiment. Notably, at 24 hpi complexes I–IV of the electron transport chain were upregulated, whereas complex V transcripts were downregulated blocking the final step of electron transport and ATP synthesis. The resulting uncoupling of the electron transport chain can lead to a process known as “uncoupling to survive” [84], which increases reactive oxygen species production and, at low levels, can boost innate immune function [85]. However, it also has the potential to lead to mitochondrial membrane permeabilisation and trigger the apoptotic pathway, which was also overrepresented at 24 hpi (Figure 3E). Collectively, these oxidoreductase alterations could form a mechanism of rapid onset of mortality in WSSV-infected shrimp by inducing apoptosis. This is further supported by the upregulation of novel miRNA Pva-pmiR-44 at 24 hpi (logFC = 1.09, FDR = 5.00 × 10^−2^) (Figure 4C), which may negatively regulate the translation of its target mRNA hormone receptor 4, resulting in increased apoptosis [86]. The concomitant significant upregulation of known miRNA Pva-miR-190 (Figure 4C) at 24 and 36 hpi (logFC = 1.20, FDR = 2.03 × 10^−3^ and logFC = 1.73, FDR = 3.44 × 10^−7^, respectively), which has previously been associated with the activation of apoptosis and positive regulation of phagocytic activity, also supports this hypothesis [24].

#### 3.5.7. Late Infection Highlights Novel WSSV-Induced Transcript and miRNA Alterations

During the later stages of WSSV infection (24 hpi onwards) several distinct processes, some of which have not previously been reported, were altered in response to WSSV injection. This included enrichment of the iron ion binding term (24 hpi) (Figure 3D), novel downregulation of glutamate and acetylcholine receptors at 24 hpi, and alterations at 36 hpi to methylation-associated transcripts such as methionine synthase-like (logFC = 3.58, FDR = 2.35 × 10^−18^) and betaine-homocysteine S-methyltransferase 1-like (logFC = −7.43, FDR = 7.89 × 10^−7^). However, the ability to interpret the impact of these changes on the late infection process is compounded by the high probability that many of the transcriptional changes at this stage are associated with shrimp that are succumbing to advanced disease.

During the WSSV infection process, few immune transcripts were differentially expressed, resulting in no enrichment of immune-related GO terms, suggesting that the immune responses mounted by whiteleg shrimp may be insufficient to prevent progression of WSSV infection to disease in this host. Whilst novel upregulated miRNAs that are predicted to target WSSV transcripts, (such as Pva-pmiR-118 (12 h; logFC = 2.20, FDR = 3.66 × 10^−2^) (Figure 4C)—predicted to target WSSV transcript Swssvgp111), may modulate WSSV transcription to defend against infection, long-term adaptive-like immune memory may be impaired, thereby counteracting these subtle effects. For example, novel shrimp miRNA Pva-pmiR-78 expression was significantly increased at 24 hpi (logFC = 7.85, FDR = 8.07 × 10^−4^) and coincided with a significant decrease in expression of its target transcript down syndrome cell adhesion molecule (Dscam)-like protein 1 homolog at 36 hpi (logFC = −2.10, FDR = 5.82 × 10^−7^) (Figure 4C,D). Dscam has been linked with pathogen recognition and playing an essential role in arthropod immunity [87], including WSSV defence and survival [88].

## 4. Discussion

**Figure 5:**
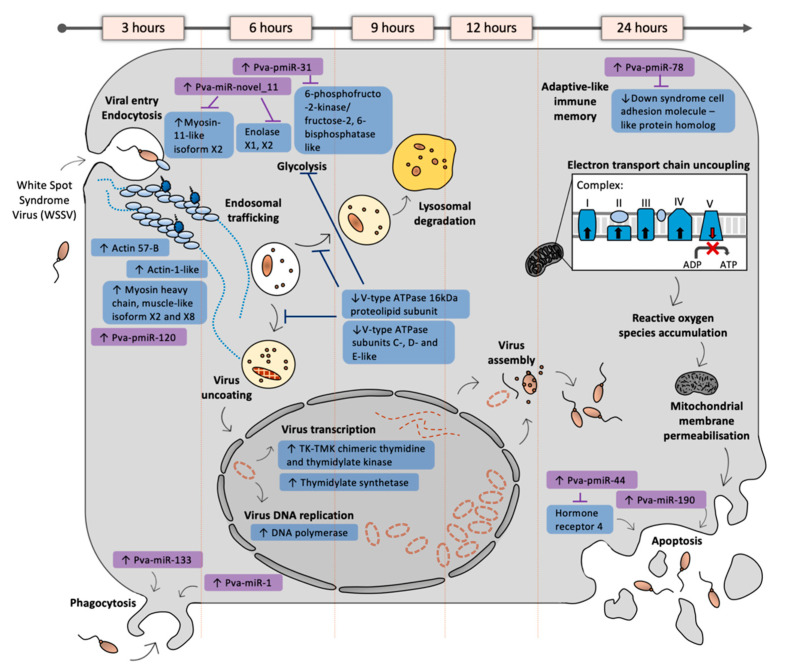
Diagram representing the transcriptome changes documented in *P. vannamei* over time following WSSV injection. Key mRNAs are represented in blue boxes and miRNAs in purple boxes, black text indicates the key processes that are altered during WSSV infection. mRNAs and miRNAs that were significantly upregulated or downregulated are marked with upward and downward pointing arrows respectively.

We investigated transcriptional responses to WSSV in the major farmed shrimp *P. vannamei* in order to establish a detailed molecular understanding of the earliest stages of WSSV infection. WSSV injection resulted in rapid temporal transcriptional (mRNA and miRNA) changes, corresponding to the various stages of infection over time. We provide the first detailed overview of the molecular mechanisms of WSSV infection and *P. vannamei* response during early infection (from 3–12 hpi), which are summarised in Figure 5. We identify novel processes associated with WSSV infection (including the upregulation of cytoskeletal components from 3–6 hpi, novel host miRNAs regulating metabolism at 6 hpi, disruption of vesicle acidification and cellular recycling at 9 hpi, and a WSSV-responsive novel host miRNA that targets an immune priming transcript at 24 hpi) that could be further explored for the development of WSSV prophylactics and therapeutics.

### 4.1. Temporal Dynamics in Transcriptional Responses to WSSV Infection Illustrate Mechanisms of Viral Entry and Replication during Early Infection, and Host Responses during the Late Infection Stages

Interrogation of the transcriptome highlighted the involvement of host cytoskeletal machinery to aid WSSV entry from 3 hpi. WSSV entry occurs via multiple endocytic routes [89,90,91], each requiring adaptations to the cytoskeleton structure for successful infection. To this end, the significant upregulation of actins may enhance WSSV entry as actin coimmunoprecipitates with VP26 and is thus a probable receptor for WSSV [92]. Following entry, actin upregulation also leads to the formation of stress fibres, providing additional molecular highways for the trafficking of the virus, similar to ebolavirus [93], and promoting phagocytosis, which may further aid entry and movement throughout the densely packed intracellular environment. The significant upregulation in myosin described here, also previously reported in the proteomes of WSSV-infected shrimp and crabs [94,95] indicates an increase in ATP-dependent molecular motors, which, when bound to actin, regulate movement within the cell [96]. However, contrary to its proposed involvement in shrimp immunity [97,98], as myosins are important factors related to growth performance [18], its hypothesised role in WSSV entry and trafficking may explain the high level of WSD susceptibility of *P. vannamei* that have been selectively bred for fast growth [99]. During late WSSV infection (48–96 hpi), a contrasting decrease in actins and myosins was reported in the hepatopancreas [100], which may also explain tissue-specific differences in susceptibility as the gills are a main target for WSSV infection. From 6 hpi, enhanced cytoskeletal processes were coupled with the upregulation of WSSV transcripts, such as WSSV thymidylate synthetase, that may offer the virus independence from host transcriptional machinery, and the enrichment of DNA-replication associated GO terms, such as deoxyribonucleotide biosynthetic process, are in line with prior literature stating viral replication begins at 6 hpi [101].

We report for the first time the involvement of 12 V-type ATPase subunits in the WSSV infection process as early infection progresses (9 hpi). V-type ATPases have been implicated as important mediators of the infection process of many viral infections including human Cytomegalovirus where they facilitate virion assembly [102] and during Influenza A virus where decreased glucose levels result in disassembly of V-ATPases, inhibition of glycolysis and Influenza A suppression [103]. Their role during WSSV infection is not presently clear, but subversion of lysosome formation could either aid or hinder the virus life cycle by protecting it from degradation in the lysosome or benefiting the host by limiting available acidic environments required by WSSV for uncoating [90]. As this response occurs following WSSV translation and replication initiation it is likely to have a limited protective effect and could indicate a response by the host to limit glycolysis, which is induced during infection [83].

In the later ‘early’ timepoints sampled (12 hpi) and at the start of ‘late’ infection (24 hpi), a shift to increased transcription occurred, coinciding with the appearance of WSD pathognomonic signs in some shrimp and the onset of mortality. In late infection (from 24 hpi) as shrimp succumbed to WSD, significant disruption to the electron transport chain was observed as components of the complexes I–IV were significantly upregulated and those of complex V, the ATPase complex, were significantly downregulated, inhibiting ATP synthesis. We hypothesise that increased reactive oxygen species were likely produced due to the high proton motive force generated, which can enhance invertebrate immunity at low levels [85]. This has previously been observed transiently from 30 min to 2 h following WSSV injection, following which WSSV displays an unusual ability to neutralise the host’s ROS defences until 24 hpi [104]. However, increased oxidative stress at 24 hpi may also lead to mitochondrial permeabilisation and initiation of apoptotic cascades [105] intended to protect the host, but instead triggering the release of newly assembled WSSV. Alterations to mitochondrial bioenergetics typically favours virus production [106]. The disappearance of mitochondrial cristae in WSSV-infected red claw crayfish gills [107] supports the hypothesis that these responses likely occur in response to WSSV infection stress as a last resort to enhance survival, but instead aid WSSV release.

### 4.2. miRNA Responses to WSSV Infection Suggest Dysregulation of Phagocytosis, Cellular Respiration and Host Immunity

Typically, increased miRNA transcription equates to posttranscriptional silencing of target mRNAs, although exceptions exist and the extent of silencing can range from fine-tuning to complete repression [108]. The significant upregulation of shrimp miRNAs reported here therefore suggests a suppressive effect of WSSV-responsive miRNAs on host transcriptional responses. During early infection, miRNAs Pva-miR-133 and Pva-miR-1 were hypothesised to have opposing effects on phagocytic activity. The former was also upregulated in sea cucumber *Apostichopus japonicus* in response to *Vibrio splendidus* causing significant increases in phagocytic activity [109], whilst the latter, a known WSSV-responsive miRNA [70] has a confirmed negative regulatory role in phagocytosis [110]. The differential regulation of phagocytic activity during the critical timepoints where virus entry occurs may indicate that this pathway is being utilised by WSSV to reach the nucleus or that the process of pathogen removal is being altered by the host to suppress WSSV infection. 

Many of the identified miRNAs were not significantly homologous to known model invertebrate miRNAs and require classification in future studies. This includes novel miRNAs Pva-pmiR-31 and Pva-miR-novel-11 whose negative regulation of glycolytic enzymes during early WSSV infection (6 hpi) coincides with Warburg-like metabolic shifts in WSSV-infected hosts [82,83] suggesting their involvement in either the effectors or suppressors of metabolic hijack by WSSV. During late infection, the ability of novel miRNA Pva-pmiR-78 to negatively regulate the translation of a Dscam-like homology provided the first suggestion that WSSV may be able to alter longer-term immune memory in WSSV-infected shrimp, in addition to shorter-term immune adaptations that enable its propagation. Dscam, a hypervariable protein that can be alternatively spliced to function as a pathogen recognition molecule for immune priming in Crustacea [111], is predicted to encode a possible 8970 unique isoforms in *P. vannamei* [112]. Knockdown of Dscam by siRNA was previously shown to decrease host survival following WSSV infection [88] and it is hypothesised that persistent WSSV exposure results in “clouds” of Dscam isoforms to defend against reinfection [112,113]. Its significant downregulation reported here, possibly because of Pva-pmiR-78 upregulation, may prevent the ability of the host to recognise WSSV in subsequent infections, increasing *P. vannamei* susceptibility to WSD.

### 4.3. Limitations

Despite the wealth of novel host–pathogen interactions described herein, the information gleaned from transcriptional studies of non-model organisms such as *P. vannamei* is still limited due to the quality of the annotations available for incomplete genomic resources. Interpretations rely heavily on the assignment of correct annotations to transcripts and miRNAs based on sequence similarity to distant species, and are underpinned by the assumption that function is evolutionarily conserved. To counteract against this, in this study we employed a number of strategies designed to improve annotations while minimising false positives, including: assigning lower thresholds for annotation, screening sequences for conserved motifs, and using complete non-redundant protein databases to query sequences. In addition, whilst miRNA target prediction typically results in long lists of candidate mRNA targets, which can be informative to identify the most likely processes affected by the regulation of those miRNAs, these lists are likely to include a large number of false positive interactions. To minimise the reporting of false positive predictions of miRNA–mRNA interactions, a combination of prediction tools was applied as recommended best practice [114]. Conservative parameters such as strict alignment in the seed region and a low minimum free energy score cut-off [115] were incorporated to further reduce this effect. Despite this, we acknowledge that the possibility of false positives (as well as false negatives) exists and that the data we present constitute hypotheses for the functionality of the interactions predicted to occur, with further experimental validation required in order to validate the proposed interactions and their biological significance.

## 5. Conclusions

We addressed a significant gap in our understanding of the early responses of susceptible *P. vannamei* to WSSV infection through integration of both transcript and miRNA expression data. Our findings identified key molecular pathways potentially involved in the initiation of the infection including the upregulation of cytoskeletal transcripts associated with viral trafficking during the initial stages of infection followed by metabolic shifts towards glycolysis, likely coordinated via miRNA-mediated post-transcriptional regulation. This was in contrast with that observed during late infection stages (after 24 hpi), where putative mitochondrial uncoupling-induced cell death was observed. Importantly we report for the first time that during late infection the novel shrimp miRNA, Pva-pmiR-78, was induced, and this was associated with a suppression in DScam transcription, a gene involved with immune memory in crustaceans. In this manner, we propose that WSSV may cause a miRNA-induced downregulation of core elements of the immune system in shrimp, contributing to its high susceptibility to WSSV infection. Together, our findings provide novel insights on how the virus maintains unrivalled access to DNA synthesis and regulation, and metabolites to ensure successful viral entry, replication, and release, including how it might avoid the immune response of the host. Our study therefore opens new avenues to develop treatment and preventative strategies to combat WSD.

## Data Availability

The data for this project are deposited within BioProject PRJNA716175. The raw sequence data (RNA-seq and microRNA-seq) are deposited within the Sequence Read Archive (accessions: SRR14027651—SRR14027746) and the de novo assembled transcriptome is deposited in figshare (https://doi.org/10.6084/m9.figshare.14410313.v1 (accessed on 1 May 2021)).

## Supplementary Materials

The following are available online at https://www.mdpi.com/article/10.3390/v13061140/s1, Figure S1: RNA-seq bioinformatics pipeline; Figure S2: miRNA-seq bioinformatics pipeline; Figure S3: Oxidative phosphorylation pathway transcript expression in response to WSSV injection over time; Table S1: Significantly differentially expressed miRNAs in response to WSSV injection over time; Table S2: Gene ontology enrichment among significantly differentially expressed transcripts; Table S3: KEGG pathway representation; Dataset S1: Differentially expressed transcripts at 3 hpi; Dataset S2: Differentially expressed transcripts at 6 hpi; Dataset S3: Differentially expressed transcripts at 9 hpi; Dataset S4: Differentially expressed transcripts at 12 hpi; Dataset S5: Differentially expressed transcripts at 24 hpi; Dataset S6: Differentially expressed transcripts at 36 hpi; Dataset S7: Complete list of reduced-redundancy *P. vannamei* miRNAs; Dataset S8: Table of predicted mRNA targets for significantly differentially expressed miRNAs.

## Appendix A

### Appendix A.1. WSSV miRNA Analysis and Results

#### Appendix A.1.1. WSSV miRNA Analysis

Viral miRNAs were predicted following the same protocol as host miRNAs, using the WSSV-CN reference genome (Genbank Accession: AF332093.3 [61]) for mapping and prediction. Known mature WSSV miRNAs downloaded from VIRmiRNA v1.0 database (July 2019, [116]) were used as a reference dataset to guide predictions. All miRNA sequences were also subjected to NCBI BlastN v2.6.0+ BlastN-Short [71]) search against all WSSV miRNAs within the VIRmiRNA v1.0 database (July 2019, [116]) with an e-value cut-off score of 1 × 10^−5^, which was chosen to allow for the short length of these sequences and expected 100% similarity score.

The breadth of coverage of small RNA (sRNA) sequences across the WSSV genome (GenBank Accession: AF332093 [61]) was assessed using Bowtie2 v2.2.9 [62]. Samtools v1.4 [64] was used to sort and convert alignments to bam files for assessment of depth and breadth of coverage. Coverage was also visually inspected using the Integrative Genomics Viewer v2.4.17 [117].

#### Appendix A.1.2. WSSV miRNA Results

The prediction methods applied within the present study did not identify any candidate WSSV-derived miRNAs. Similarity searches of collapsed sRNA reads against 40 previously classified WSSV miRNAs within the VIRmiRNA database [116] also returned no significant hits. Given the clear exponential increase in viral mRNA transcription observed in the RNA-seq dataset, it is unusual that no WSSV-derived miRNAs were identified within the data. WSSV miRNA expression patterns have been reported in previous studies [118,119], therefore, viral miRNAs were expected to be present within the current dataset and play a role in post-transcriptional regulation during infection. However, a recent study showed that WSSV-derived sRNAs (constituted 1.4% of the sRNAs sequenced in acutely infected Chinese shrimp *P. chinensis*, and only a minor proportion (3.6%) of these were identified as potential miRNAs originating from pre-miRNA structures. The majority of WSSV sRNAs were likely to be short interfering RNAs (siRNAs) [120]. Given the low abundance of WSSV sRNAs that correspond to miRNAs, it may be that a greater sequencing depth is required to identify and quantify these miRNAs. Factors within the library preparation protocol, such as the use of a gel-free size selection protocol to purify sRNA sequences from total RNA may also have contributed to this result; with additional washes to purify the miRNA fragments possibly contributing to the loss of lowly expressed WSSV miRNAs.

Several studies have reported that the structure of viral miRNAs is similar to animal miRNAs due to the reliance on host enzymatic machinery for their production [121,122,123]. However, if their structures differ at all, for example as a result of non-canonical biogenesis pathways, this may lead to an absence of specific sequence features, such as 3′ overhangs that sequencing adapters recognise and bind to during the library preparation protocols and within prediction software. Recently, a study exploring the potential of Papillomaviruses (PVs) to produce miRNAs reported a similar lack of PV miRNAs identified using these methods, despite near-complete coverage of the virus genome sequences by sRNA reads [124]. In the present study, a similar high breadth of coverage of the WSSV genome (96.2%) was observed within the sequenced reads. After reanalysis of the data used in previous publications in support of the existence of PV miRNAs, Chirayil et al. [124] detected no PV miRNA candidates, indicating that the differences seen were due to differences in the bioinformatics analyses, such as increased sequencing error and small nucleotide polymorphism allowance leading to false alignments and downstream prediction. If PVs and WSSV are able to encode miRNAs, their sequences may therefore be non-canonical and not easily identified using presently available tools. Due to the high diversity among viral genomes, both homology-based and machine-learning algorithms perform poorly in the identification of viral miRNAs [125].

Currently, identified miRNAs derive mainly from viruses with dsDNA genomes, therefore, it is reasonable to expect that WSSV would possess the ability to produce these also. In lymphoma cells heavily infected with Kaposi’s Sarcoma-Associated Herpesvirus (KSHV), the few viral miRNAs identified accounted for at least 80% of all miRNAs [126]; therefore, it is surprising that no WSSV miRNAs were identified here. It is still debated whether other viruses such as (Human Immunodeficiency Virus) HIV-1 are able to produce their own miRNAs (reviewed by Balasubramaniam, Pandhara and Dash [127]) due to the lack of suitable protocols for their identification. Crucially, miRNAs require experimental validation prior to deposition within publicly available databases and many viral miRNAs currently lack this. WSSV miRNA sequencing studies rarely present evidence of the ability of these to fold into bifurcated hairpin precursor structures [118,120], which is required evidence when establishing *bona fide* miRNAs. Therefore, in the absence of experimental evidence confirming the existence of WSSV-derived miRNAs, it remains unclear if this virus generates miRNA during infection in our test species.
