# Supplementary material for: Global mRNA and miRNA Analysis Reveal Key Processes in the Initial Response to Infection with WSSV in the Pacific Whiteleg Shrimp
Source: Viruses. 2021 Jun 13;13(6):1140. doi: 10.3390/v13061140 (PMC8231841; doi:10.3390/v13061140)
Supplement: Supplementary file 1 [file viruses-13-01140-s001.zip › supplementary.pdf]

Supplementary Material

Figure S1: RNA-seq analysis workflow. Detailed bioinformatics workflow (black boxes) for analysis of RNA-seq dataset. For each step, specific tools, scripts and parameters utilised are depicted within orange boxes and a summary of the resulting outputs are displayed in blue boxes.

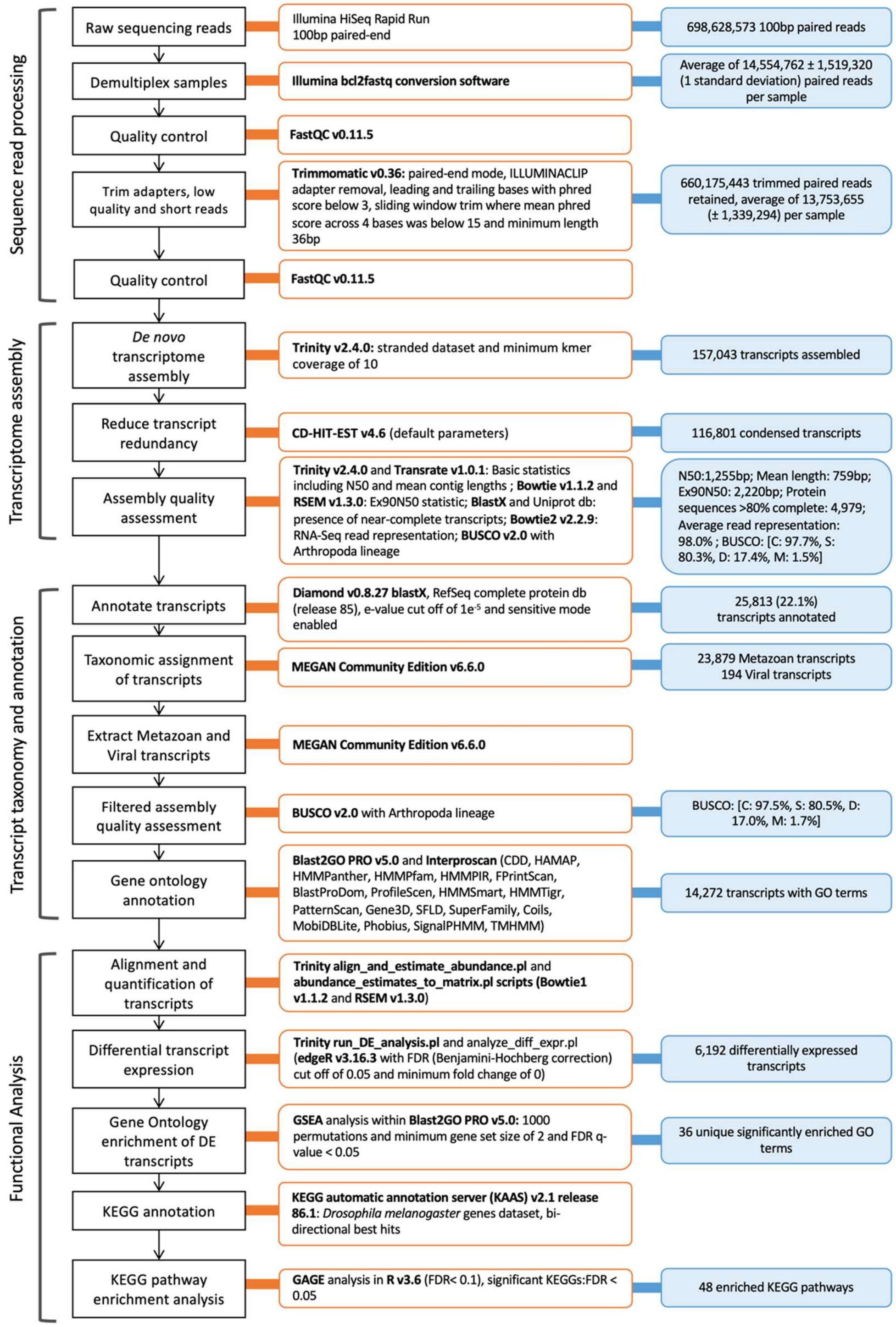

Figure S2: miRNA-seq analysis workflow. Detailed bioinformatics workflow (black boxes) for analysis of microRNA-seq dataset. For each step, specific tools, scripts and parameters utilised are depicted within orange boxes and a summary of the resulting outputs are displayed in blue boxes.

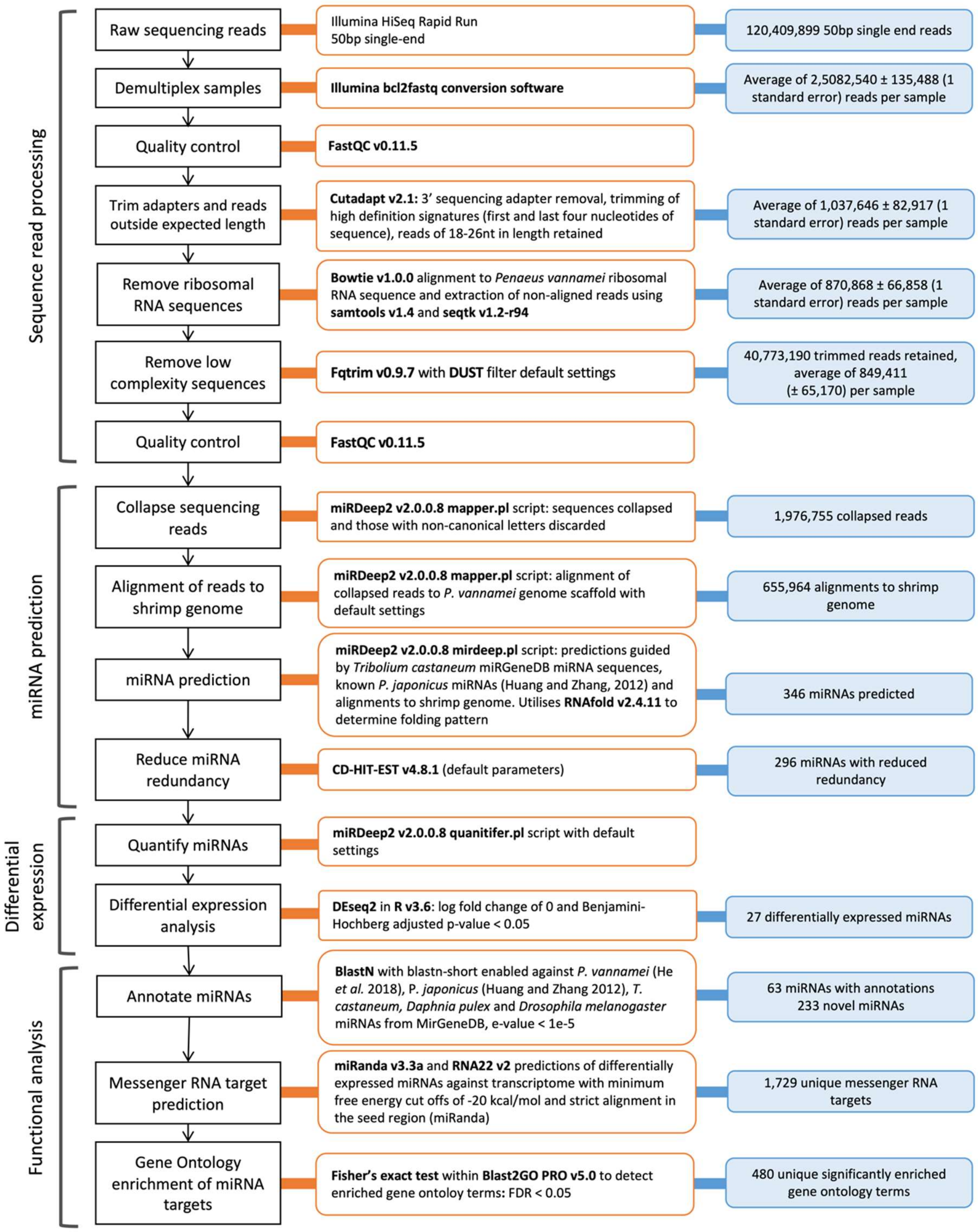

Figure S3: Significantly over and underrepresented oxidative phosphorylation KEGG pathway. Oxidative phosphorylation KEGG pathway (*Drosophila melanogaster;* ID: 00190) populated with log fold change transcription data, using the Pathview package in R, from shrimp at **(a)** 3h, **(b)** 6h, **(c)** 9h, **(d)** 12h, **(e)** 24h and **(f)** 36h post WSSV injection. Upregulated transcripts are shown in red and downregulated transcripts are shown in blue. Transcripts present within the *Drosophila melanogaster* pathway, that were unchanged between WSSV-injected and time-matched control shrimp at each time point are shaded grey and transcripts absent from the dataset are indicated by white boxes. Transcripts that were significantly differentially expressed are outlined with a thick black box. The time course of expression data indicates that at 9 hpi elements of all oxidative phosphorylation complexes are downregulated resulting in increased oxidative stress. At 12 hpi, there is some evidence of recovery as several elements of these complexes are upregulated. Whilst this upregulation is observed in a larger number of complex subunits from 24 hpi, there is evidence of electron transport chain uncoupling as elements of complex I-IV are upregulated and complex V are downregulated.

(a)

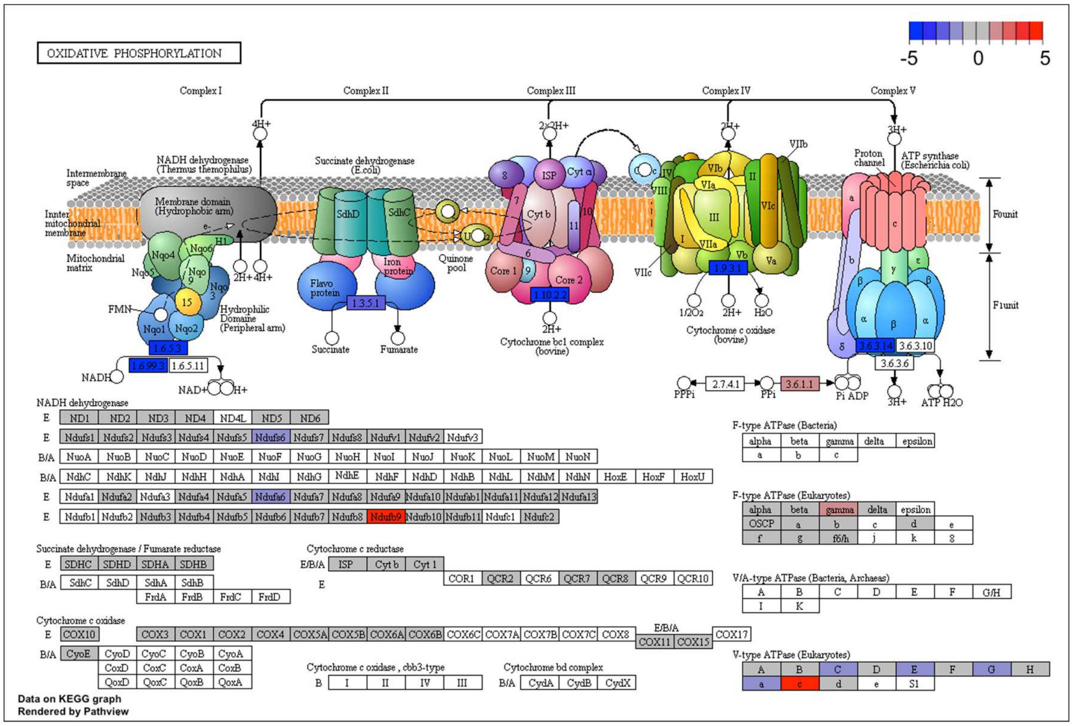
OXIDATIVE PHOSPHORYLATION
-5 0 5
Complex I
Complex II
Complex III
Complex IV
Complex V
NADH dehydrogenase (Thermus themophilus)
Succinate dehydrogenase (E.coli)
Cytochrome bc1 complex (bovine)
Cytochrome c oxidase (bovine)
Proton channel
ATP synthase (Escherichia coli)
Intermembrane space
Inner mitochondrial membrane
Mitochondrial matrix
Membrane domain (Hydrophobic arm)
Hydrophilic Domain (Peripheral arm)
Quinone pool
Flavo protein
Iron protein
Succinate
Fumarate
F0 unit
F1 unit
NADH dehydrogenase
Succinate dehydrogenase / Fumarate reductase
Cytochrome c reductase
Cytochrome c oxidase
Cytochrome c oxidase, cbb3-type
Cytochrome bd complex
F-type ATPase (Bacteria)
F-type ATPase (Eukaryotes)
V/A-type ATPase (Bacteria, Archaeas)
V-type ATPase (Eukaryotes)
Data on KEGG graph
Rendered by Pathview

(b)

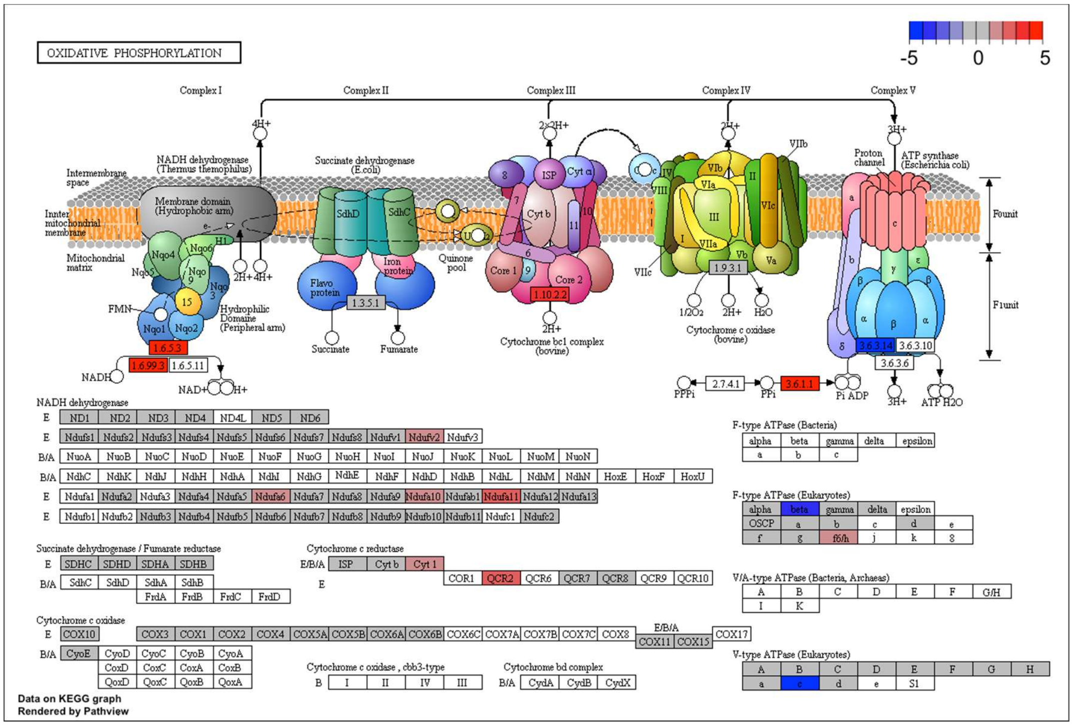
OXIDATIVE PHOSPHORYLATION
-5 0 5
Complex I
Complex II
Complex III
Complex IV
Complex V
NADH dehydrogenase (Thermus themophilus)
Succinate dehydrogenase (E.coli)
Cytochrome bc1 complex (bovine)
Cytochrome c oxidase (bovine)
Proton channel
ATP synthase (Escherichia coli)
Intermembrane space
Inner mitochondrial membrane
Mitochondrial matrix
Membrane domain (Hydrophobic arm)
Hydrophilic Domain (Peripheral arm)
Quinone pool
Flavo protein
Iron protein
Succinate
Fumarate
F0 unit
F1 unit
NADH dehydrogenase
Succinate dehydrogenase / Fumarate reductase
Cytochrome c reductase
Cytochrome c oxidase
Cytochrome c oxidase, cbb3-type
Cytochrome bd complex
F-type ATPase (Bacteria)
F-type ATPase (Eukaryotes)
V/A-type ATPase (Bacteria, Archaeas)
V-type ATPase (Eukaryotes)
Data on KEGG graph
Rendered by Pathview

(c)

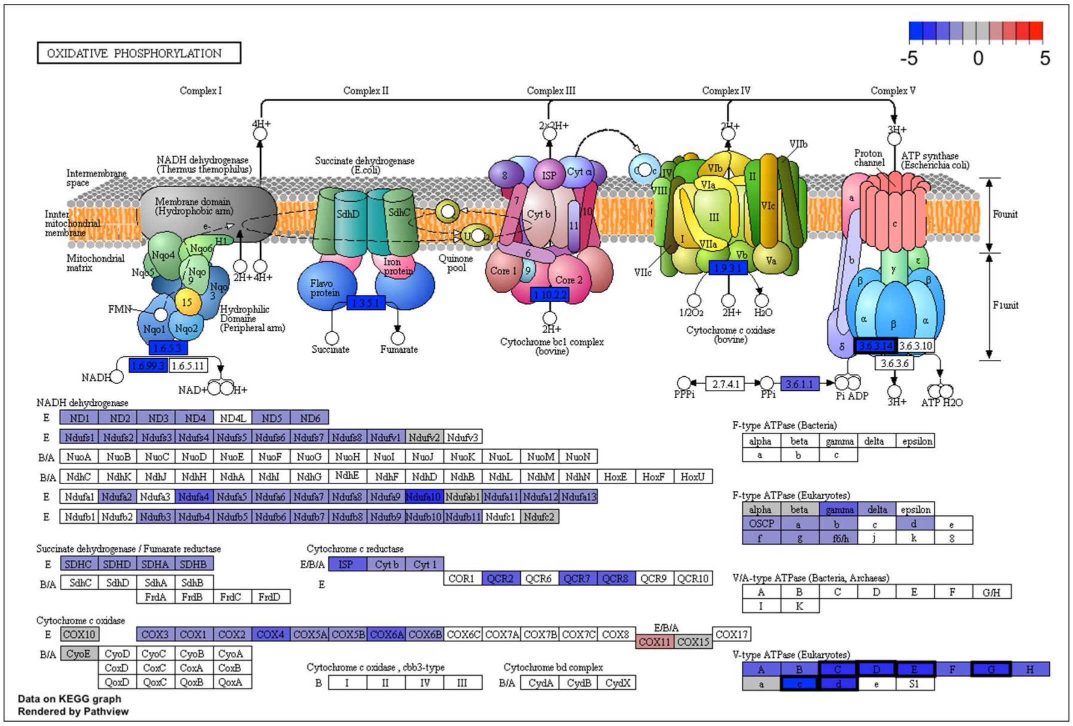

OXIDATIVE PHOSPHORYLATION
-5 0 5
Complex I
Complex II
Complex III
Complex IV
Complex V
NADH dehydrogenase (Thermus themophilus)
Succinate dehydrogenase (E.coli)
Cytochrome bc1 complex (bovine)
Cytochrome c oxidase (bovine)
ATP synthase (Escherichia coli)
Intermembrane space
Inner mitochondrial membrane
Mitochondrial matrix
Membrane domain (Hydrophobic arm)
Hydrophilic Domain (Peripheral arm)
Quinone pool
Proton channel
F0 unit
F1 unit
1.6.5.3
1.6.99.3
1.6.5.11
1.3.5.1
1.10.2.2
1.9.3.1
3.6.3.14
3.6.3.10
3.6.3.6
2.7.4.1
3.6.1.1
NADH
NAD+
Succinate
Fumarate
PPi
Pi ADP
ATP H2O
NADH dehydrogenase
Succinate dehydrogenase / Fumarate reductase
Cytochrome c reductase
Cytochrome c oxidase
Cytochrome c oxidase , cbb3-type
Cytochrome bd complex
F-type ATPase (Bacteria)
F-type ATPase (Eukaryotes)
V/A-type ATPase (Bacteria, Archaeas)
V-type ATPase (Eukaryotes)
Data on KEGG graph
Rendered by Pathview

(d)

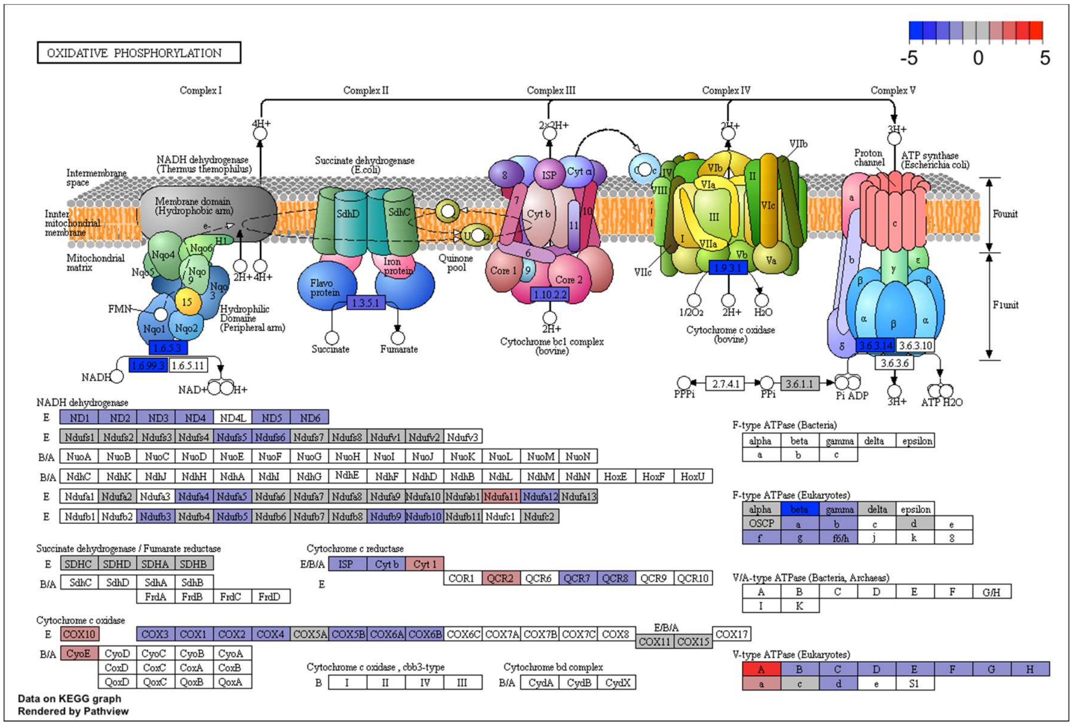

OXIDATIVE PHOSPHORYLATION
-5 0 5
Complex I
Complex II
Complex III
Complex IV
Complex V
NADH dehydrogenase (Thermus themophilus)
Succinate dehydrogenase (E.coli)
Cytochrome bc1 complex (bovine)
Cytochrome c oxidase (bovine)
ATP synthase (Escherichia coli)
1.6.5.3
1.6.99.3
1.6.5.11
1.3.5.1
1.10.2.2
1.9.3.1
3.6.3.14
3.6.3.10
3.6.3.6
2.7.4.1
3.6.1.1
NADH dehydrogenase
Succinate dehydrogenase / Fumarate reductase
Cytochrome c reductase
Cytochrome c oxidase
Cytochrome c oxidase , cbb3-type
Cytochrome bd complex
F-type ATPase (Bacteria)
F-type ATPase (Eukaryotes)
V/A-type ATPase (Bacteria, Archaeas)
V-type ATPase (Eukaryotes)
Data on KEGG graph
Rendered by Pathview

(e)

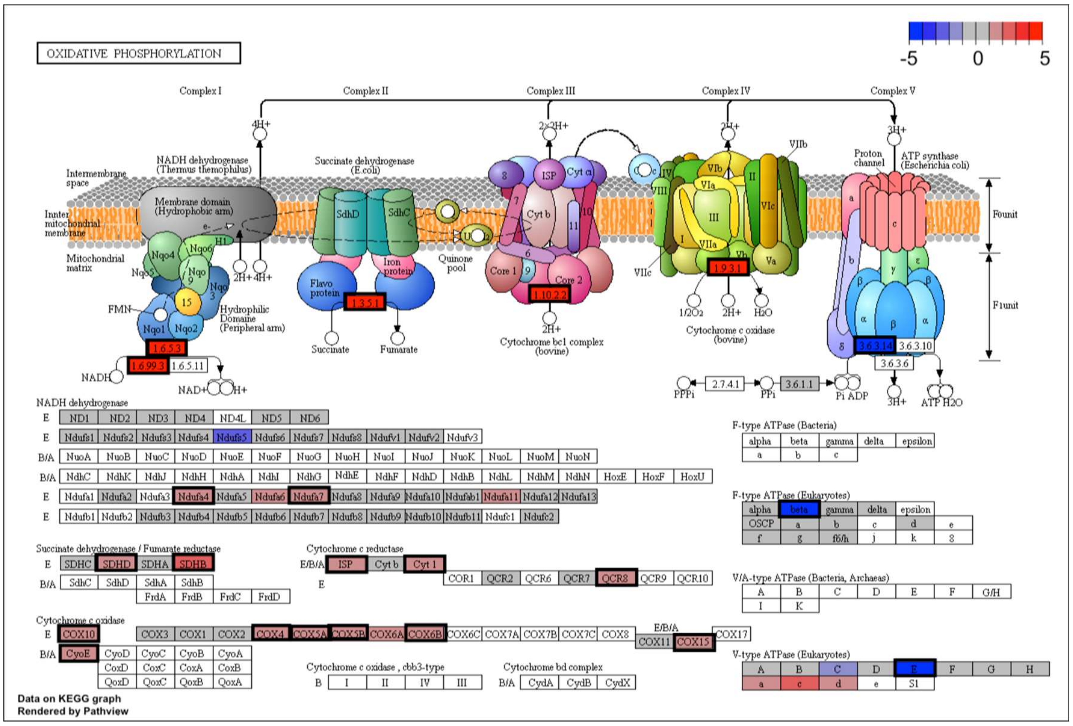
OXIDATIVE PHOSPHORYLATION
-5
0
5
Complex I
Complex II
Complex III
Complex IV
Complex V
NADH dehydrogenase (Thermus themophilus)
Succinate dehydrogenase (E.coli)
Cytochrome bc1 complex (bovine)
Cytochrome c oxidase (bovine)
ATP synthase (Escherichia coli)
Proton channel
Fo unit
F1 unit
Intermembrane space
Inner mitochondrial membrane
Mitochondrial matrix
Membrane domain (Hydrophobic arm)
Hydrophilic Domain (Peripheral arm)
Quinone pool
Succinate
Fumarate
NADH dehydrogenase
Succinate dehydrogenase / Fumarate reductase
Cytochrome c reductase
Cytochrome c oxidase
Cytochrome c oxidase, cbb3-type
Cytochrome bd complex
F-type ATPase (Bacteria)
F-type ATPase (Eukaryotes)
V/A-type ATPase (Bacteria, Archaeas)
V-type ATPase (Eukaryotes)
Data on KEGG graph
Rendered by Pathview

(f)

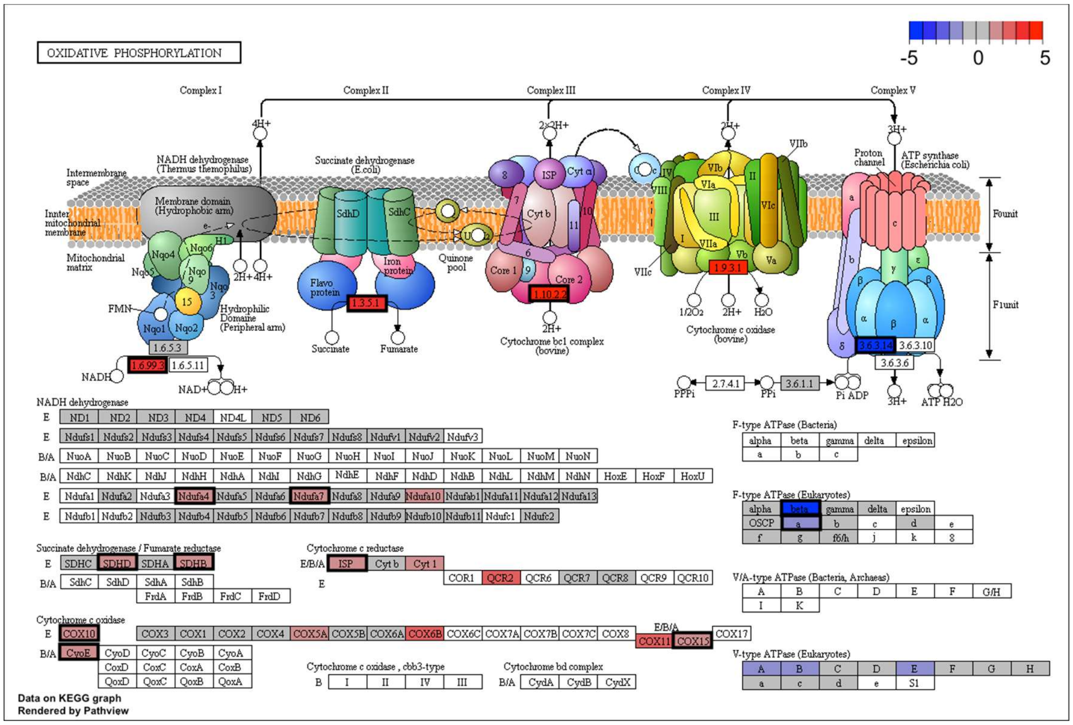
OXIDATIVE PHOSPHORYLATION
-5
0
5
Complex I
Complex II
Complex III
Complex IV
Complex V
NADH dehydrogenase (Thermus themophilus)
Succinate dehydrogenase (E.coli)
Cytochrome bc1 complex (bovine)
Cytochrome c oxidase (bovine)
ATP synthase (Escherichia coli)
Proton channel
Fo unit
F1 unit
Intermembrane space
Inner mitochondrial membrane
Mitochondrial matrix
Membrane domain (Hydrophobic arm)
Hydrophilic Domain (Peripheral arm)
Quinone pool
Succinate
Fumarate
NADH dehydrogenase
Succinate dehydrogenase / Fumarate reductase
Cytochrome c reductase
Cytochrome c oxidase
Cytochrome c oxidase, cbb3-type
Cytochrome bd complex
F-type ATPase (Bacteria)
F-type ATPase (Eukaryotes)
V/A-type ATPase (Bacteria, Archaeas)
V-type ATPase (Eukaryotes)
Data on KEGG graph
Rendered by Pathview

Table S1. Significantly differentially expressed miRNAs in response to WSSV injection over time. Differentially expressed miRNAs (adjusted p-value < 0.05), separated by experimental time point, in response to WSSV injection. Upregulated miRNAs are highlighted in green and downregulated miRNAs are highlighted in red.

Time post injection	miRNA	annotation	sequence	log2FC	p-adj
3h	LVANscaffold_2661_16907	Pva-pmiR-120	TTCTCCAGTAGCCTGTTAG	22.654	3.45E-07
3h	LVANscaffold_1752_10269	Pva-miR-133	TTGGTCCCCTTCAACCAGCTGT	6.415	2.17E-03
6h	LVANscaffold_1752_10269	Pva-miR-133	TTGGTCCCCTTCAACCAGCTGT	7.997	1.76E-05
6h	LVANscaffold_1495_8360	Pva-pmiR-31	ATTATCATTCTTTGGCGTCCGG	1.740	1.71E-04
6h	LVANscaffold_3588_23974	Pva-miR-1	TGGAATGTAAAGAAGTATGGAG	2.699	1.43E-03
6h	LVANscaffold_2877_18478	Pva-miR-750	CCAGATCTAACTCTTCCAGCTCA	-1.999	9.83E-03
6h	LVANscaffold_1646_9393	Pva-miR-novel_11	TCCCTGAGACCCTTTCTTGTGA	0.977	2.43E-02
6h	LVANscaffold_2658_16858	Pva-pmiR-118	CCATTACCTTCTTCCCCTCTT	2.195	3.66E-02
12h	LVANscaffold_3085_20010	Pva-pmiR-141	GCAGTGCATCACCTCCCTAGGC	2.678	8.79E-04
12h	LVANscaffold_1304_7171	Pva-pmiR-18	CTTGGGAGGTGCTGCACTGCTA	1.199	4.83E-02
24h	LVANscaffold_2088_12726	Pva-pmiR-81	TGACTAGATACGACACTCATCCA	6.888	1.65E-12
24h	LVANscaffold_2088_12722	Pva-pmiR-80	TATCACAGTTGAAGTTACCTAGA	6.148	2.23E-07
24h	LVANscaffold_2088_12713	Pva-pmiR-78	TTAGGAACTGACACTGTGGTAGG	7.851	8.07E-04
24h	LVANscaffold_2088_12728	Pva-mir-36	TCACCGGGTAGGATTCATCCAA	7.511	2.03E-03
24h	LVANscaffold_3170_20854	Pva-miR-190	AGATATGTTTGATATTCTTGGTTG	1.201	2.03E-03
24h	LVANscaffold_2789_17819	Pva-miR-193b	CAATGCCCTTGGAAATCCCAAA	5.311	5.69E-03
24h	LVANscaffold_2088_12712	Pva-pmiR-77	TGACTAGATCCAACACTCATCCAG	6.241	1.16E-02
24h	LVANscaffold_4587_26117	Pva-miR-79a	ATAAAGCTAGATTACCAAAGTC	-0.834	1.16E-02
24h	LVANscaffold_1304_7171	Pva-pmiR-18	CTTGGGAGGTGCTGCACTGCTA	1.144	1.48E-02
24h	LVANscaffold_4587_26123	Pva-miR-79b	ATAAAGCTAGATTACCAAAGCA	-0.866	2.26E-02
24h	LVANscaffold_1654_9453	Pva-pmiR-44	AGGCAAGACTCCGGCGTAGCTG	1.800	4.27E-02
24h	LVANscaffold_2888_18595	Pva-pmiR-133	CTTACGACCATCTAGCACGGCGC	1.091	5.00E-02
24h	LVANscaffold_2127_13165	Pva-miR-279	TGACTAGATCCACACTCATCCA	-0.327	9.76E-02
36h	LVANscaffold_2088_12726	Pva-pmiR-81	TGACTAGATACGACACTCATCCA	6.223	2.02E-08
36h	LVANscaffold_3170_20854	Pva-miR-190	AGATATGTTTGATATTCTTGGTTG	1.728	3.44E-07
36h	LVANscaffold_2088_12722	Pva-pmiR-80	TATCACAGTTGAAGTTACCTAGA	6.538	3.07E-06
36h	LVANscaffold_1304_7171	Pva-pmiR-18	CTTGGGAGGTGCTGCACTGCTA	1.600	8.30E-05
36h	LVANscaffold_2661_16907	Pva-pmiR-120	TTCTCCAGTAGCCTGTTAG	16.669	5.27E-04
36h	LVANscaffold_4587_26117	Pva-miR-79a	ATAAAGCTAGATTACCAAAGTC	-0.991	8.33E-04
36h	LVANscaffold_1262_6936	Pva-pmiR-15	CTTCCCTTCGTTGTTCCTGTTG	4.860	1.53E-03
36h	LVANscaffold_2088_12713	Pva-pmiR-78	TTAGGAACTGACACTGTGGTAGG	7.164	1.53E-03
36h	LVANscaffold_4587_26123	Pva-miR-79b	ATAAAGCTAGATTACCAAAGCA	-0.988	3.70E-03
36h	LVANscaffold_2088_12712	Pva-pmiR-77	TGACTAGATCCAACACTCATCCAG	6.042	1.22E-02
36h	LVANscaffold_1567_8839	Pva-pmiR-36	CCAACGATAGAAAGGTGTCCTC	0.901	1.48E-02
36h	LVANscaffold_2088_12728	Pva-mir-36	TCACCGGGTAGGATTCATCCAA	6.089	1.54E-02
36h	LVANscaffold_3018_19452	Pva-pmiR-138	CCCCATCGTGATAAGCCCCTAT	2.367	3.72E-02

Time post injection	miRNA	annotation	sequence	log2FC	p-adj
36h	LVANscaffold_505_1551	Pva-miR-novel_12	ATTACGACCTTCTAGCACGG	1.188	3.88E-02
36h	LVANscaffold_2657_16836	Pva-pmiR-116	TAGCACCAGTGGATTCAGCATG	-0.508	4.37E-02
36h	LVANscaffold_268_845	Pva-pmiR-121	TATTATGCTAAGATTCGTGTAT	1.806	4.87E-02

Table S2. GO term enrichment among differentially expressed transcripts. Table of enriched gene ontology terms determined by gene set enrichment analysis (GSEA) within Blast2GO PRO v5.0 at 3, 6, 9, 12, 24 and 36 hours post WSSV determined using lists of differentially expressed transcripts for each time point post infection. GO terms that were not significantly enriched (FDR q value > 0.05) are highlighted in grey.

Time post injection	GO ID	GO Name	GO Category	Size	Enrichment score	FDR q value
3h	GO:0005856	cytoskeleton	CC	47	-0.345	2.41E-03
3h	GO:0015629	actin cytoskeleton	CC	39	-0.354	3.21E-03
3h	GO:0044430	cytoskeletal part	CC	39	-0.354	5.11E-03
3h	GO:0030016	myofibril	CC	7	-0.683	5.13E-03
3h	GO:0005861	troponin complex	CC	7	-0.683	5.48E-03
3h	GO:0044449	contractile fiber part	CC	7	-0.683	5.52E-03
3h	GO:0036379	myofilament	CC	7	-0.683	5.67E-03
3h	GO:0043292	contractile fiber	CC	7	-0.683	5.75E-03
3h	GO:0099080	supramolecular complex	CC	7	-0.683	5.96E-03
3h	GO:0030017	sarcomere	CC	7	-0.683	6.48E-03
3h	GO:0099512	supramolecular fiber	CC	7	-0.683	6.63E-03
3h	GO:0005865	striated muscle thin filament	CC	7	-0.683	6.71E-03
3h	GO:0007010	cytoskeleton organization	BP	5	-0.766	7.21E-03
3h	GO:0099081	supramolecular polymer	CC	7	-0.683	7.28E-03
3h	GO:0016459	myosin complex	CC	32	-0.304	3.97E-02
3h	GO:0003774	motor activity	MF	36	-0.277	7.02E-02
6h	GO:0036338	viral membrane	CC	20	-0.556	0.00E+00
6h	GO:0019031	viral envelope	CC	20	-0.556	0.00E+00
6h	GO:0019012	virion	CC	20	-0.556	0.00E+00
6h	GO:0044423	virion part	CC	20	-0.556	0.00E+00
6h	GO:0015629	actin cytoskeleton	CC	58	-0.300	8.60E-04
6h	GO:0003774	motor activity	MF	57	-0.301	9.50E-04
6h	GO:0016459	myosin complex	CC	49	-0.313	1.11E-03
6h	GO:0044430	cytoskeletal part	CC	60	-0.277	1.15E-03
6h	GO:0005856	cytoskeleton	CC	75	-0.235	3.91E-03
6h	GO:0009262	deoxyribonucleotide metabolic process	BP	3	-0.969	9.40E-03
6h	GO:0009263	deoxyribonucleotide biosynthetic process	BP	3	-0.969	9.93E-03
6h	GO:0016874	ligase activity	MF	3	-0.922	1.41E-02
6h	GO:0006220	pyrimidine nucleotide metabolic process	BP	2	-0.973	7.29E-02
6h	GO:0009265	2'-deoxyribonucleotide biosynthetic process	BP	2	-0.973	7.35E-02

Time post injection	GO ID	GO Name	GO Category	Size	Enrichment score	FDR q value
6h	GO:0006221	pyrimidine nucleotide biosynthetic process	BP	2	-0.973	7.50E-02
6h	GO:0009219	pyrimidine deoxyribonucleotide metabolic process	BP	2	-0.973	7.61E-02
6h	GO:0072527	pyrimidine-containing compound metabolic process	BP	2	-0.973	7.78E-02
6h	GO:0072528	pyrimidine-containing compound biosynthetic process	BP	2	-0.973	7.94E-02
6h	GO:0046385	deoxyribose phosphate biosynthetic process	BP	2	-0.973	7.95E-02
6h	GO:0009221	pyrimidine deoxyribonucleotide biosynthetic process	BP	2	-0.973	8.05E-02
6h	GO:0019692	deoxyribose phosphate metabolic process	BP	2	-0.973	8.10E-02
6h	GO:0044877	protein-containing complex binding	MF	10	-0.475	8.39E-02
6h	GO:0009394	2'-deoxyribonucleotide metabolic process	BP	2	-0.973	8.54E-02
6h	GO:0051015	actin filament binding	MF	10	-0.475	8.67E-02
9h	GO:0019031	viral envelope	CC	27	-0.714	0.00E+00
9h	GO:0036338	viral membrane	CC	27	-0.714	0.00E+00
9h	GO:0044423	virion part	CC	28	-0.681	0.00E+00
9h	GO:0019012	virion	CC	28	-0.681	0.00E+00
9h	GO:0043492	ATPase activity, coupled to movement of substances	MF	4	0.851	3.56E-02
9h	GO:0042623	ATPase activity, coupled	MF	4	0.851	4.00E-02
9h	GO:1901137	carbohydrate derivative biosynthetic process	BP	5	-0.731	4.31E-02
9h	GO:0015405	P-P-bond-hydrolysis-driven transmembrane transporter activity	MF	4	0.851	4.75E-02
9h	GO:0065009	regulation of molecular function	BP	3	0.877	4.79E-02
9h	GO:0090662	ATP hydrolysis coupled transmembrane transport	BP	10	0.512	4.80E-02
9h	GO:0015630	microtubule cytoskeleton	CC	4	0.772	4.81E-02
9h	GO:0015078	proton transmembrane transporter activity	MF	10	0.512	4.91E-02
9h	GO:0098660	inorganic ion transmembrane transport	BP	10	0.512	4.96E-02
9h	GO:0009263	deoxyribonucleotide biosynthetic process	BP	3	-0.921	5.10E-02
9h	GO:0009262	deoxyribonucleotide metabolic process	BP	3	-0.921	5.19E-02
9h	GO:0099132	ATP hydrolysis coupled cation transmembrane transport	BP	10	0.512	5.21E-02
9h	GO:0015988	energy coupled proton transmembrane	BP	10	0.512	5.22E-02

Time post injection	GO ID	GO Name	GO Category	Size	Enrichment score	FDR q value
		transport, against electrochemical gradient				
9h	GO:0030695	GTPase regulator activity	MF	5	0.678	5.29E-02
9h	GO:0098796	membrane protein complex	CC	10	0.512	5.32E-02
9h	GO:0098662	inorganic cation transmembrane transport	BP	10	0.512	5.36E-02
9h	GO:0022853	active ion transmembrane transporter activity	MF	3	0.850	5.43E-02
9h	GO:0016887	ATPase activity	MF	4	0.851	5.54E-02
9h	GO:0060589	nucleoside-triphosphatase regulator activity	MF	5	0.678	5.57E-02
9h	GO:0006812	cation transport	BP	10	0.512	5.68E-02
9h	GO:0046961	proton-transporting ATPase activity, rotational mechanism	MF	3	0.850	5.68E-02
9h	GO:0060089	molecular transducer activity	MF	10	0.477	5.79E-02
9h	GO:0042625	ATPase coupled ion transmembrane transporter activity	MF	3	0.850	5.84E-02
9h	GO:0016757	transferase activity, transferring glycosyl groups	MF	4	0.734	5.85E-02
9h	GO:0019829	cation-transporting ATPase activity	MF	3	0.850	6.03E-02
9h	GO:0098655	cation transmembrane transport	BP	10	0.512	6.06E-02
9h	GO:0004871	signal transducer activity	MF	10	0.477	6.11E-02
9h	GO:0036442	proton-exporting ATPase activity	MF	3	0.850	6.19E-02
9h	GO:0008047	enzyme activator activity	MF	5	0.678	6.24E-02
9h	GO:0030234	enzyme regulator activity	MF	8	0.623	6.27E-02
9h	GO:0033176	proton-transporting V-type ATPase complex	CC	9	0.511	6.34E-02
9h	GO:1901565	organonitrogen compound catabolic process	BP	7	0.551	6.42E-02
9h	GO:0015672	monovalent inorganic cation transport	BP	10	0.512	6.43E-02
9h	GO:0098772	molecular function regulator	MF	11	0.448	6.46E-02
9h	GO:0005096	GTPase activator activity	MF	5	0.678	6.58E-02
9h	GO:0015991	ATP hydrolysis coupled proton transport	BP	10	0.512	6.98E-02
9h	GO:0016469	proton-transporting two-sector ATPase complex	CC	10	0.512	7.00E-02
9h	GO:0015077	monovalent inorganic cation transmembrane transporter activity	MF	12	0.429	7.07E-02
9h	GO:0034220	ion transmembrane transport	BP	12	0.429	7.24E-02

Time post injection	GO ID	GO Name	GO Category	Size	Enrichment score	FDR q value
9h	GO:1902600	proton transmembrane transport	BP	10	0.512	7.61E-02
9h	GO:0022890	inorganic cation transmembrane transporter activity	MF	12	0.429	8.07E-02
9h	GO:0099131	ATP hydrolysis coupled ion transmembrane transport	BP	10	0.512	8.43E-02
9h	GO:0042626	ATPase activity, coupled to transmembrane movement of substances	MF	4	0.851	9.23E-02
12h	GO:0019012	virion	CC	30	-0.742	0.00E+00
12h	GO:0019031	viral envelope	CC	29	-0.740	0.00E+00
12h	GO:0036338	viral membrane	CC	29	-0.740	0.00E+00
12h	GO:0044423	virion part	CC	30	-0.742	0.00E+00
24h	GO:0019012	virion	CC	30	-0.960	0.00E+00
24h	GO:0019031	viral envelope	CC	29	-0.960	0.00E+00
24h	GO:0044423	virion part	CC	30	-0.960	0.00E+00
24h	GO:0036338	viral membrane	CC	29	-0.960	0.00E+00
24h	GO:0005230	extracellular ligand-gated ion channel activity	MF	18	-0.456	4.01E-02
24h	GO:0005506	iron ion binding	MF	47	-0.287	4.50E-02
24h	GO:0016705	oxidoreductase activity, acting on paired donors, with incorporation or reduction of molecular oxygen	MF	45	-0.280	6.47E-02
24h	GO:0016646	oxidoreductase activity, acting on the CH-NH group of donors, NAD or NADP as acceptor	MF	4	-0.876	6.59E-02
36h	GO:0044423	virion part	CC	30	-0.973	0.00E+00
36h	GO:0019012	virion	CC	30	-0.973	0.00E+00
36h	GO:0036338	viral membrane	CC	29	-0.973	0.00E+00
36h	GO:0019031	viral envelope	CC	29	-0.973	0.00E+00
36h	GO:1901605	alpha-amino acid metabolic process	BP	29	-0.368	2.50E-02
36h	GO:0044272	sulfur compound biosynthetic process	BP	10	-0.591	4.39E-02

Table S3. KEGG pathway representation. Table of significantly (q value < 0.05) enriched KEGG pathways at 3, 6, 9, 12, 24 and 36 hours post white spot syndrome virus infection in *Penaeus vannamei* gills. Drosophila melanogaster homologs were retrieved for differentially expressed transcripts at each time points by BlastN search against the uniprot database and KEGG pathway annotations retrieved using the KEGG Automatic Annotation Server (KAAS) v2.1 (release 86.1, May 2018) against the *D. melanogaster* genes dataset with bidirectional best hits (Moriya et al., 2007). Enrichment analysis was performed using the Generally Applicable Gene-set Enrichment for Pathway Analysis (GAGE) v3.9 (Luo et al., 2009) package in R v3.3.2 (R Core Team, 2017). Overrepresented pathways are highlighted in green and underrepresented pathways are highlighted in red.

Time post injection	KEGG ID	KEGG Pathway	Over or under representation	p value	q value	Transcripts in pathway
3h	dme03008	Ribosome biogenesis in eukaryotes	OVER	4.23E-07	3.97E-05	53
3h	dme00190	Oxidative phosphorylation	UNDER	8.83E-12	8.30E-10	83
6h	dme03010	Ribosome	OVER	3.26E-12	2.96E-10	113
6h	dme00190	Oxidative phosphorylation	OVER	1.17E-06	5.31E-05	83
6h	dme04145	Phagosome	OVER	9.24E-04	2.80E-02	40
6h	dme00970	Aminoacyl-tRNA biosynthesis	OVER	1.56E-03	3.56E-02	34
6h	dme00520	Amino sugar and nucleotide sugar metabolism	OVER	4.42E-03	8.04E-02	31
9h	dme00240	Pyrimidine metabolism	OVER	3.64E-04	2.15E-02	54
9h	dme03008	Ribosome biogenesis in eukaryotes	OVER	4.78E-04	2.15E-02	53
9h	dme00190	Oxidative phosphorylation	UNDER	2.56E-29	2.30E-27	83
9h	dme04145	Phagosome	UNDER	4.78E-06	2.15E-04	38
12h	dme03008	Ribosome biogenesis in eukaryotes	OVER	1.62E-11	1.49E-09	53
12h	dme03013	RNA transport	OVER	1.85E-05	8.52E-04	88
12h	dme00240	Pyrimidine metabolism	OVER	1.76E-04	7.28E-04	52
12h	dme04144	Endocytosis	OVER	2.06E-04	4.73E-03	83
12h	dme03020	RNA polymerase	OVER	1.05E-03	1.91E-02	19
12h	dme00970	Aminoacyl-tRNA biosynthesis	OVER	1.25E-03	1.91E-02	34
12h	dme03018	RNA degradation	OVER	2.48E-03	3.26E-02	40
12h	dme00190	Oxidative phosphorylation	UNDER	3.30E-14	3.04E-12	83
12h	dme03010	Ribosome	UNDER	6.16E-07	2.83E-05	113

Time post injection	KEGG ID	KEGG Pathway	Over or under representation	p value	q value	Transcripts in pathway
24h	dme03050	Proteasome	OVER	2.37E-17	2.13E-15	32
24h	dme04141	Protein processing in endoplasmic reticulum	OVER	4.13E-13	1.86E-11	83
24h	dme03008	Ribosome biogenesis in eukaryotes	OVER	1.98E-05	5.93E-04	53
24h	dme04215	Apoptosis - multiple species	OVER	8.52E-04	1.92E-02	11
24h	dme03013	RNA transport	OVER	1.18E-03	2.13E-02	87
24h	dme03020	RNA polymerase	OVER	1.96E-03	2.68E-02	19
24h	dme00190	Oxidative phosphorylation	OVER	2.13E-03	2.68E-02	83
24h	dme03060	Protein export	OVER	2.38E-03	2.68E-02	15
24h	dme03010	Ribosome	OVER	3.06E-03	3.06E-02	113
24h	dme03040	Spliceosome	OVER	4.45E-03	4.00E-02	98
24h	dme00240	Pyrimidine metabolism	OVER	5.37E-03	4.39E-02	52
24h	dme03030	DNA replication	UNDER	3.39E-10	2.80E-08	30
24h	dme04146	Peroxisome	UNDER	6.22E-10	2.80E-08	50
24h	dme00071	Fatty acid degradation	UNDER	6.85E-07	2.06E-05	21
24h	dme00280	Valine, leucine and isoleucine degradation	UNDER	1.34E-05	2.58E-04	30
24h	dme00980	Metabolism of xenobiotics by cytochrome P450	UNDER	1.71E-05	2.58E-04	10
24h	dme00380	Tryptophan metabolism	UNDER	1.72E-05	2.58E-04	13
24h	dme00410	beta-Alanine metabolism	UNDER	3.09E-05	3.97E-04	11
24h	dme00040	Pentose and glucuronate interconversions	UNDER	4.12E-05	3.97E-04	16
24h	dme01212	Fatty acid metabolism	UNDER	6.24E-05	5.89E-04	27
24h	dme00640	Propanoate metabolism	UNDER	6.54E-05	5.89E-04	17
24h	dme00982	Drug metabolism - cytochrome P450	UNDER	1.00E-04	8.20E-04	10
24h	dme00500	Starch and sucrose metabolism	UNDER	1.54E-04	1.16E-03	12
24h	dme00310	Lysine degradation	UNDER	6.21E-04	4.30E-03	29
24h	dme00630	Glyoxylate and dicarboxylate metabolism	UNDER	8.60E-04	5.53E-03	23
24h	dme00350	Tyrosine metabolism	UNDER	1.26E-03	7.57E-03	10
24h	dme04391	Hippo signaling pathway - fly	UNDER	1.69E-03	9.53E-03	41
24h	dme04068	FoxO signaling pathway	UNDER	2.12E-03	1.12E-02	38
24h	dme03410	Base excision repair	UNDER	4.31E-03	2.15E-02	15
24h	dme04392	Hippo signaling pathway -multiple species	UNDER	5.50E-03	2.61E-02	13
24h	dme00620	Pyruvate metabolism	UNDER	6.02E-03	2.70E-02	21

Time post injection	KEGG ID	KEGG Pathway	Over or under representation	p value	q value	Transcripts in pathway
24h	dme00511	Other glycan degradation	UNDER	6.30E-03	2.70E-02	12
24h	dme00010	Glycolysis / Gluconeogenesis	UNDER	7.00E-03	2.86E-02	26
24h	dme00250	Alanine, aspartate and glutamate metabolism	UNDER	7.45E-03	2.91E-02	12
24h	dme00062	Fatty acid elongation	UNDER	9.34E-03	3.50E-02	13
24h	dme04142	Lysosome	UNDER	1.54E-02	5.21E-02	56
24h	dme00260	Glycine, serine and threonine metabolism	UNDER	1.57E-02	5.21E-02	22
24h	dme03440	Homologous recombination	UNDER	1.62E-02	5.21E-02	16
24h	dme00531	Glycosaminoglycan degradation	UNDER	1.67E-02	5.21E-02	12
24h	dme04150	mTOR signaling pathway	UNDER	1.68E-02	5.21E-02	55
24h	dme00514	Other types of O-glycan biosynthesis	UNDER	1.88E-02	5.63E-02	10
24h	dme03430	Mismatch repair	UNDER	2.82E-02	8.18E-02	16
24h	dme04070	Phosphatidylinositol signaling system	UNDER	3.52E-02	9.89E-02	27
36h	dme03008	Ribosome biogenesis in eukaryotes	OVER	5.50E-15	5.01E-13	53
36h	dme03010	Ribosome	OVER	1.16E-11	5.29E-10	113
36h	dme03050	Proteasome	OVER	1.04E-05	2.73E-04	32
36h	dme03040	Spliceosome	OVER	1.20E-05	2.73E-04	98
36h	dme03013	RNA transport	OVER	2.14E-05	3.89E-04	88
36h	dme03020	RNA polymerase	OVER	8.55E-05	1.30E-03	19
36h	dme04141	Protein processing in endoplasmic reticulum	OVER	2.99E-03	3.88E-02	83
36h	dme00240	Pyrimidine metabolism	OVER	4.52E-03	5.14E-02	54
36h	dme03030	DNA replication	UNDER	2.20E-10	2.01E-08	30
36h	dme04146	Peroxisome	UNDER	9.20E-08	4.18E-06	50
36h	dme00280	Valine, leucine and isoleucine degradation	UNDER	2.66E-06	8.06E-05	30
36h	dme04068	FoxO signaling pathway	UNDER	5.15E-06	1.17E-04	38
36h	dme00980	Metabolism of xenobiotics by cytochrome P450	UNDER	2.45E-05	3.85E-04	10
36h	dme00380	Tryptophan metabolism	UNDER	2.54E-05	3.85E-04	13
36h	dme00410	beta-Alanine metabolism	UNDER	3.20E-05	4.16E-04	12
36h	dme00982	Drug metabolism - cytochrome P450	UNDER	8.23E-05	8.97E-04	10
36h	dme00071	Fatty acid degradation	UNDER	8.87E-05	8.97E-04	21
36h	dme00640	Propanoate metabolism	UNDER	2.02E-04	1.84E-03	17
36h	dme00310	Lysine degradation	UNDER	4.34E-04	3.59E-03	27

Time post injection	KEGG ID	KEGG Pathway	Over or under representation	p value	q value	Transcripts in pathway
36h	dme00040	Pentose and glucuronate interconversions	UNDER	6.45E-04	4.89E-03	16
36h	dme01212	Fatty acid metabolism	UNDER	1.00E-03	7.03E-03	27
36h	dme03430	Mismatch repair	UNDER	1.30E-03	8.44E-03	16
36h	dme03440	Homologous recombination	UNDER	2.21E-03	1.34E-02	18
36h	dme03410	Base excision repair	UNDER	4.51E-03	2.57E-02	15
36h	dme00062	Fatty acid elongation	UNDER	4.86E-03	2.60E-02	14
36h	dme00630	Glyoxylate and dicarboxylate metabolism	UNDER	5.78E-03	2.92E-02	23
36h	dme00620	Pyruvate metabolism	UNDER	7.05E-03	3.38E-02	21
36h	dme04150	mTOR signaling pathway	UNDER	7.89E-03	3.59E-02	55
36h	dme00250	Alanine, aspartate and glutamate metabolism	UNDER	1.08E-02	4.67E-02	14
36h	dme04142	Lysosome	UNDER	1.38E-02	5.71E-02	56
36h	dme00010	Glycolysis / Gluconeogenesis	UNDER	2.31E-02	9.12E-02	26
